# Unification of Cas protein families and a simple scenario for the origin and evolution of CRISPR-Cas systems

**DOI:** 10.1186/1745-6150-6-38

**Published:** 2011-07-14

**Authors:** Kira S Makarova, L Aravind, Yuri I Wolf, Eugene V Koonin

**Affiliations:** 1 National Center for Biotechnology Information, National Library of Medicine, National Institutes of Health, 8600 Rockville Pike, Bethesda, MD 20894, USA

## Abstract

**Background:**

The CRISPR-Cas adaptive immunity systems that are present in most Archaea and many Bacteria function by incorporating fragments of alien genomes into specific genomic loci, transcribing the inserts and using the transcripts as guide RNAs to destroy the genome of the cognate virus or plasmid. This RNA interference-like immune response is mediated by numerous, diverse and rapidly evolving Cas (CRISPR-associated) proteins, several of which form the Cascade complex involved in the processing of CRISPR transcripts and cleavage of the target DNA. Comparative analysis of the Cas protein sequences and structures led to the classification of the CRISPR-Cas systems into three Types (I, II and III).

**Results:**

A detailed comparison of the available sequences and structures of Cas proteins revealed several unnoticed homologous relationships. The Repeat-Associated Mysterious Proteins (RAMPs) containing a distinct form of the RNA Recognition Motif (RRM) domain, which are major components of the CRISPR-Cas systems, were classified into three large groups, Cas5, Cas6 and Cas7. Each of these groups includes many previously uncharacterized proteins now shown to adopt the RAMP structure. Evidence is presented that large subunits contained in most of the CRISPR-Cas systems could be homologous to Cas10 proteins which contain a polymerase-like Palm domain and are predicted to be enzymatically active in Type III CRISPR-Cas systems but inactivated in Type I systems. These findings, the fact that the CRISPR polymerases, RAMPs and Cas2 all contain core RRM domains, and distinct gene arrangements in the three types of CRISPR-Cas systems together provide for a simple scenario for origin and evolution of the CRISPR-Cas machinery. Under this scenario, the CRISPR-Cas system originated in thermophilic Archaea and subsequently spread horizontally among prokaryotes.

**Conclusions:**

Because of the extreme diversity of CRISPR-Cas systems, in-depth sequence and structure comparison continue to reveal unexpected homologous relationship among Cas proteins. Unification of Cas protein families previously considered unrelated provides for improvement in the classification of CRISPR-Cas systems and a reconstruction of their evolution.

**Open peer review:**

This article was reviewed by Malcolm White (nominated by Purficacion Lopez-Garcia), Frank Eisenhaber and Igor Zhulin. For the full reviews, see the Reviewers' Comments section.

## Background

The CRISPR-Cas is an adaptive immunity system that is present in most archaea and many bacteria, and functions on a "Lamarckian inheritance" principle. The CRISPR-Cas loci in prokaryote genomes consist of an array of direct, typically palindromic repeats known as CRISPR (Clustered Regularly Interspaced Short Palindrome Repeats) and unique spacers located between the CRSIPR repeats [1]. The CRISPR repeat arrays are usually associated with *cas *(CRISPR-associated) genes which encode proteins with a variety of predicted nucleic acid-manipulating activities such as nucleases, helicases and polymerases [1,2]. The striking feature of the CRISPR loci is that some of the unique spacers in the CRISPR repeat cassettes are identical to fragments of viral (bacteriophage) genes [3,4]. This finding together with the predicted activities of the Cas proteins prompted the hypothesis that CRISPR-Cas is a system of adaptive immunity that integrates short genomic segments of selfish elements (viruses or plasmids) into specific loci in prokaryotic genomes and then employs these inserts to abrogate the replication of the cognate agents via a RNAi-like mechanism [5].

These predictions attracted the attention of several research groups resulting in considerable experimental support for the above hypothesis [6]. These studies have elucidated many important details of the molecular mechanisms of the CRISPR-Cas systems and the three distinct functional stages of their operation [7,8]. During the first stage, adaptation, short pieces of DNA (characteristic length of approximately 30 bp) homologous to virus or plasmid sequences (known as proto-spacers) are integrated into the CRISPR loci [6,9,10]. The short (3 or 4 nucleotides) proto-spacer adjacent motifs (PAMs) located immediately downstream of the proto-spacer appear to determine the selection of the protospacer followed by integration into a pre-existing CRISPR array [11,12]. The second stage, expression and processing, involves transcription and cleavage of long primary transcript of a CRISPR locus (pre-crRNA) that is processed into short crRNAs. This step is catalyzed by endoribonucleases encoded by the *cas *genes that either operate as a subunit of a larger complex (e.g. Cascade, CRISPR-associated complex for antiviral defense in *Escherichia coli *[13]) or as a stand-alone enzyme, e.g., Cas6 in the archaeon *Pyrococcus furiosus *[14,15]. At the third stage, interference, the alien nucleic acid (DNA or RNA) is targeted by a ribonucleoprotein complex containing a crRNA guide and a set of Cas proteins, and cleaved within or in the vicinity of the PAM sequence [7,9,10,16]. In several CRISPR-Cas systems, crRNA have been shown to be complementary to either strand of the phage or plasmid which is best compatible with DNA being the target [6,17]. Direct demonstration of DNA being the target of the CRISPR-Cas machinery has come from experiments in *Staphylococcus epidermidis*. In this case, insertion of a self-splicing intron into the proto-spacer sequence of the target gene rendered the respective plasmid resistant to the CRISPR-mediated immunity [18]. Recently, the *E. coli *Cascade complex containing crRNA has been shown to recognize the target DNA, with the specificity defined by the crRNA sequence, and displace the non-complementary strand in an energy-independent manner [8]. However, *in vitro *experiments with one of the CRISPR-Cas systems (Type IIIB, formerly known as Cmr system or RAMP module) from the archaeon *P. furiosus *showed that the crRNA rather targets the mRNA [15]; it remains to be determined whether this is also the case *in vivo *or the *P. furiosus *CRISPR-Cas systems target alien DNA in addition to or instead of mRNA. In any case, these findings emphasize the remarkable mechanistic and functional diversity of the CRISPR-Cas systems.

The apparent functional diversity of CRISPR-Cas systems is paralleled by the equally notable diversity of Cas proteins: at least 45 distinct protein families have been identified in association with CRISPR loci in various bacterial and archaeal genomes [19]. An analysis involving more sensitive methods of sequence comparison and additional evidence from genomic context has revealed distant homologous relationships between several of these families, suggesting that even more Cas protein families might be linked subsequently thanks to the growth of genomic and structural data sets and further advances in computational analysis [5].

The recently updated classification of CRISPR-Cas systems divides them into three distinct types (I, II and III) [20]. All these systems contain two universal genes: *cas1*, a metal-dependent DNAse with no sequence specificity that could be involved in the integration of the alien DNA (spacer) into CRISPR cassettes [21,22], and *cas2*, a metal-dependent endoribonuclease, that also appears to be involved in the spacer acquisition stage [23]. Otherwise, the three types of CRISPR-Cas systems substantially differ in their sets of constituent genes, and each is characterized, respectively, by a unique signature gene. The signature genes for the three types are, respectively, *cas3 *(a superfamily 2 helicase containing an N-terminal HD superfamily nuclease domain) [24]), *cas9 *(a large protein containing a predicted RuvC-like and HNH nuclease domains) and *cas10 *(a protein containing a domain homologous the palm domain of nucleic acid polymerases and nucleotide cyclases) [20]. Within these three types, CRISPR-Cas systems can be further classified into subtypes based on a number of considerations that include distinct signature genes along with the phylogeny of the universal *cas1 *gene [20]. The Cas proteins known as RAMPs (Repeat-Associated Mysterious Proteins) are present in several copies in both type I and III systems. Some of the RAMPs have been shown to possess sequence- or structure-specific RNAse activity that is involved in the processing of pre-crRNA transcripts [13,14,17]. The crystal structures of several RAMPs have been solved and indicate that they contain one or two domains which display distinct versions of the RNA recognition motif (RRM) or ferredoxin fold [5,16,17,25,26]. The experimentally characterized activities and functions of the key Cas proteins are listed in Table 1.

**Table 1:** Experimentally characterized and predicted functions of the core components of CRISPR-Cas systems

Family	Experimental/*in silico *evidence	Prediction
**Cas1**	Metal-dependent deoxyribonuclease; a unique fold consisting of a N-terminal β strand domain and a C-terminal α-helical domain [21,68]; also binds RNA [21,68]	Involved in integration of spacer DNA into CRISPR repeats.

**Cas2**	RNAse specific to U-rich regions [23]	Facilitates spacer selection and/or integration. Could be involved in further crRNA cleavage.

**Cas3** **(helicase and HD domain)**	Single-stranded DNA nuclease (HD domain) and ATP-dependent helicase [24]; required for interference [13].	Cuts DNA during interference; promotes strand separation.

**Stand alone HD nuclease**	Metal-dependent deoxyribonuclease specific for double-stranded oligonucleotides [69].	Cuts DNA during interference.

**Cas4**	RecB-like nuclease homolog with three-cysteine C-terminal cluster [5]	Might be involved in spacer acquisition

**Cas5**	RAMP [5], subunit of Cascade complex [8,13].	Might substitute for Cas6 if catalytically active. Otherwise might be involved in both interference and adaptation stages.

**Cas6**	RAMP [5]; metal-independent endoribonuclease that generates crRNAs, subunit of Cascade complex [8,13-15,17].	

**Cas7**	RAMP [5], subunit of Cascade complex [13]; present Cascade complex of I-E systems in 6 copies [8] and in several copies in I-A systems [16].	Implicated in interference; binds crRNA; if enzymatically active, might be involved in RNA-guided RNA cleavage.

**Cas8** **(large subunit)**	Subunit of Cascade complex [13].	Inactivated Cas10 polymerase-like protein, binds DNA, interacts with HD domain and a RAMP carrying crRNA; could be involved in both interference and spacer selection stages.

**Cas10** **(large subunit, CRISPR polymerase)**	Subunit of Cascade (Cmr) complex [15]; homologous to Palm domain polymerases and cyclases.	Same as Cas8, but fused to HD and thus cuts ssDNA; might be involved in strand separation.

**Small subunit**	Small, mostly alpha helical protein, subunit of Cascade complex [13,15]; present in Cascade complex of I-E systems in two copies [8]	Specifically binds DNA; might recognize PAM.

Despite the recent progress in understanding the relationships between various Cas proteins, over 30 Cas protein families apparently do not display similarity with each other [20]. Here we present an analysis of these proteins families that reveals several previously unnoticed relationships. We then use the results of this analysis to develop an evolutionary scenario for the origin of the CRISPR-Cas systems and to propose hypotheses on some of the key aspects of CRISPR-Cas function.

## Results and discussion

### The RAMPs

#### Cas7 represents a distinct major group of RAMPs

**Figure 1:**
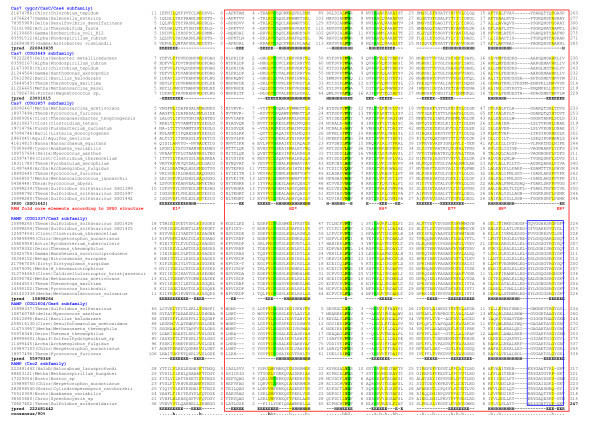
**Multiple alignment of Cas7 subfamilies and related families of RAMPs**. The multiple sequence alignment includes the conserved blocks identified by HHpred (red box), secondary structure predictions and the secondary structure elements extracted from the crystal structure of the Cas7 from *S. solfataricus *[16]. Secondary structure prediction showed as follows: 'H' indicates α-helix, 'E' indicates extended conformation (β-strand). The sequences are denoted by their GI numbers and species names. G-rich loop region of RAMPs is shown by blue box. The positions of the first and the last residues of the aligned region in the corresponding protein are indicated for each sequence. The numbers within the alignment represent poorly conserved inserts that are not shown. The coloring is based on the consensus shown underneath the alignment; 'h' indicates hydrophobic residues (WFYMLIVACTH), 'p' indicates polar residues (EDKRNQHTS), 's' indicates small residues (ACDGNPSTV).

Cas7 (COG1857) is present in most of the type I CRISPR-Cas systems. Previously, members of this family have been confidently identified in the I-A, I-B, I-C, I-E systems [5]. As part of the recent update of the classification of CRISPR-Cas systems, we performed exhaustive sequence database searches for all Cas protein families using the HHpred profile against profile search method [27]. These searches revealed statistically significant similarity between Cas7 family proteins and various RAMP families [for example, the search with the query sequence ST0029 of the Cas7 (formerly known as DevR/Csa2 family) from *Sulfolobus tokodaii*, identifies the TIGR02581 profile for SSO1426 (or Csm3, COG1337) family RAMP with a probability of 0.93 and many other RAMP families with lower scores]. The reciprocal search started from the SSO1426 protein sequence hits the PFAM profile PF01905 which corresponds to the Cas7 family (probability 0.97). We used the alignments obtained during these and other searches started from other query sequences along with secondary structure predictions to construct multiple alignments for Cas7 and a number of most closely related RAMP subfamilies (Figure 1). In all these proteins, HHpred identifies several conserved blocks including the characteristic glycine-rich loop, based on both secondary structure and sequence conservation (Figure 1). In addition, the N-terminal beta strand (the first strand of the RRM fold) that is an essential structural feature of the RAMPs (Additional File 1) could be identified based on the secondary structure prediction. In the Cas7 group RAMP typical of Type I CRISPR-Cas systems, the signature glycine-rich (G-rich) loop of RAMPs is notably eroded. However, the characteristic structural organization of this region, namely the alpha-helix and the beta-strand that flank the glycine-rich loop at the N- and C- termini, respectively, in other RAMPs, seems to be present in these proteins. Collectively, these observations indicate that Cas7 proteins present in the I-A, I-B, I-C, and I-E CRISPR-Cas system subtypes comprise a distinct family within the RAMP superfamily.

After these analyses have been performed, the crystal structure of Cas7 (Csa2) from the Crenarchaeon *Sulfolobus solfataricus *has been reported [16]. Examination of this structure clearly demonstrates the presence of a single RAMP domain that contains four inserts within the RRM core and a C-terminal extension. None of these additional domains of Cas7 show sequence or structural similarity to any known domains [16].

#### Classification and evolution of RAMPs

**Figure 2:**
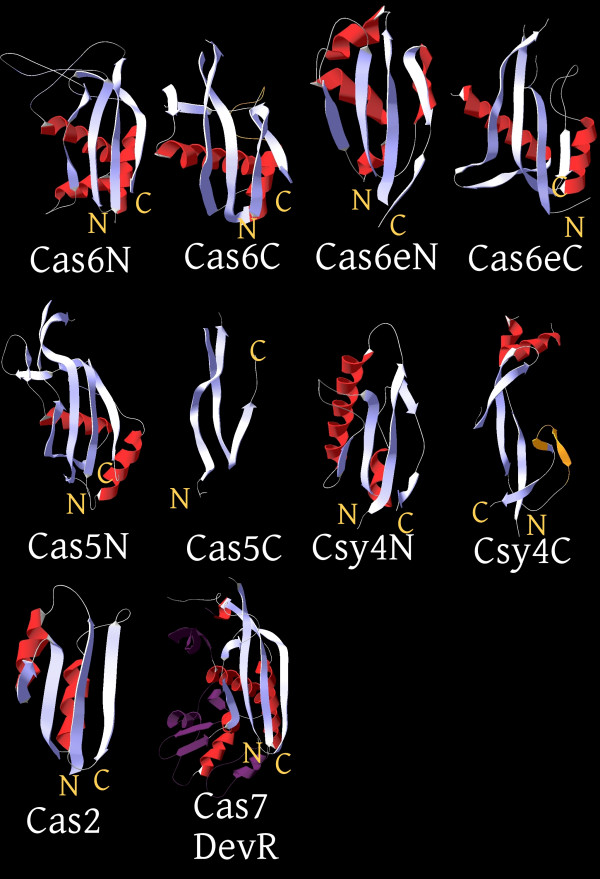
**The RRM fold of RAMPs and Cas2**. The RRM fold domains of Cas2 and the three major RAMP groups proposed in the text are shown in cartoon representation with their N- and C- termini indicated. In Cas7, the insertions into the core of the RRM fold are shown in a darker shade. In the RAMPs with two RRM fold domains, these are respectively labeled as N(-terminal) and C(-terminal). The distinct C-terminal domains of Cas5 and Cas6f (Csy4) are also shown. In Cas6f, the glycine-rich loop, which is embedded in a beta-hairpin in contrast to the typical helix-strand element, is colored orange. Note the "horizontal" packing of the first helix of the core RRM fold against the 4 strand sheet, which is one of the characteristic structural features of the RAMPs (apparent in Cas7, Cas6, Cas6e and Cas5). The following PDB ids were used to generate these representations: 2I0X (Cas2);_3PS0 (Cas7); 3I4H (Cas6); 1WJ9 (Cas6e/CasE); 3KG4(Cas5); 2XLJ (Cas6f/Csy4).

**Figure 3:**
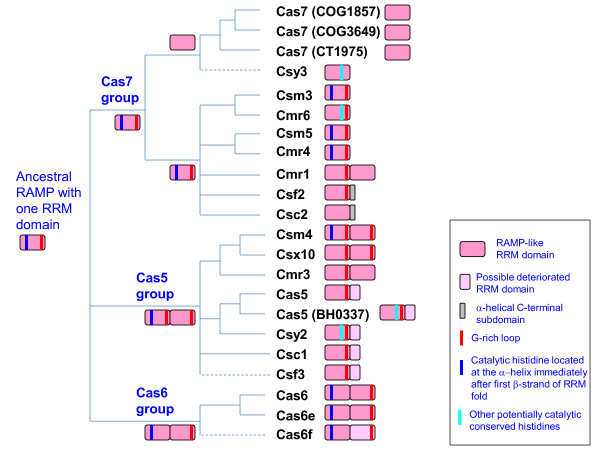
**Classification of the RAMPs**. The tree-like scheme of RAMP relationships is based on the sequence similarity, structural features and neighborhood analysis described in the text, and should not be construed as a phylogenetic tree. Unresolved relationships are shown as multifurcations and tentative assignments are shown by broken lines. The catalytic activity of some of the RAMP proteins of the Cas5 and Cas7 groups involving the partially conserved histidines shown in the figure should be considered a tentative prediction.

The demonstration that the Cas7 family belongs to the RAMP superfamily prompted us to further investigate the relationships between the RAMPs. We performed DALI searches with all available RAMP structures (Figure 2 and Additional File 2) and HHpred searches using representatives of 19 RAMP families, and collected similarity scores between the corresponding profiles (Additional File 3). For each family we predicted secondary structure or assigned secondary structure elements from the known structures of RAMPs (Additional File 1). Combining the results of these analyses, we classify the RAMP superfamily into three major groups: Cas5, Cas6 and Cas7 (Figure 3).

The Cas5 group RAMPs (Cas5/COG1688, Cmr3/COG1769, Csm4/COG1567, Csy2, Csc1) were unified on the basis of sequence similarity that in most cases was identifiable by HHpred and the presence of a C-terminal domain downstream of the G-rich loop (Figure 2). For some of these C-terminal domains, an RRM fold can be predicted (Additional File 3). For example, in the Cmr3 subfamily (Subtype III-B), the predicted secondary structure elements of the C-terminal domain are compatible with the RRM arrangement (Additional File 1). Moreover, this domain ends with a second G-rich loop whereas in the Csm4 subfamily from type III-A CRISPR-Cas systems (the closest homolog of the Cmr3 family), this loop is almost completely degraded (Additional File 1). The proteins of the Csx10 subfamily that is also related to Cmr3/Csm4, contain two predicted RRM domains followed by a clearly identifiable G-rich loop (Additional File 1). The Csx10 subfamily can be unequivocally linked to the Cas5 group and specifically to Cmr3 and Csm4 families through HHpred searches (the best hit for a representative of the family Rcas_3289 from *Roseiflexus castenholzii *is the pfam09700 profile for Cmr3 with the probability of 99%.) The remaining Cas5 proteins including Cas5 proper, Csy2, Csc1 and Csf3 contain a single N-terminal RRM domain that terminates with the G-rich loop and is followed by a distinct C-terminal beta-meander domain. Thus, the large Cas5 group of RAMPs consists of two distinct subgroups one of which contains two RRM domains and the other one contains only one RRM domain (Figure 2). It remains uncertain as to which is the ancestral form, i.e. whether the ancestor of the Cas5 group already contained two RRM domains, and the C-terminal one was lost or severely deteriorated in one of the subgroups, or the ancestral form possessed a single RRM domain that was duplicated in one of the subgroups.

The Cas6 group includes Cas6 proteins proper (COG1853/COG5551) that have been experimentally characterized as the CRISPR transcript processing RNA endonucleases [13,14,26,28] as well as highly diverged homologs from the I-E (Cas6e) and I-F (Cas6f) CRISPR-Cas subtypes. This grouping is supported by the available structures and is compatible with the reported functions for the representatives of each family. Most of the Cas6 proteins encompass two well-defined RRM domains which are connected by a "flange" in the extended conformation and have a glycine-rich loop upstream of the last strand of the second RRM fold domain. Thus, the ancestor of the Cas6 group can be confidently inferred to have possessed two RRM domains. However, the Cas6f proteins contain a typical N-terminal RRM domain and a distinct C-terminal domain that displays certain topological features reminiscent of the RRM fold (see Additional file 1 and 2) and contains a C-terminal G-rich loop but does not show significant sequence or structural similarity to any RRM domains (Figure 2). This domain could be either a grossly distorted RRM or a distinct beta-meander that convergently acquired the G-rich loop.

The Cas7 group includes Cas7 proper (COG1857) and a variety of RAMPs mostly associated with the Type III CRISPR-Cas systems. All of these proteins contain a single RRM domain with additional elaborations as demonstrated by the recently reported Cas7 structure (Figure 2), sequence comparison and secondary structure prediction. The Type III RAMP families (Csm3/COG1337 and Csm5/COG1332 in subtype III-A; Cmr6/COG1604, Cmr4/COG1336, Cmr1/COG1367 in subtype III-B; Csc2 in subtype I-D and Csf2 from the system of unknown subtype) are more similar to each other (Additional File 3) than to Cas7 but share with Cas7 a number of conserved sequence motifs (Figure 1 and Additional File 1), the overall sequence similarity identifiable by HHpred (Additional File 3) and the absence of the additional RRM domain after the G-rich loop (or its counterpart). The Csy3 subfamily is tentatively included in this group based on the secondary structure prediction (no extension after the G-rich loop compatible with another RRM domain). Some members of the Cas7 group, such as Cmr1, contain a second predicted RRM domain. Furthermore, the RAMPs of the Cas7 group show a tendency for gene duplication at least in Type III CRISPR-Cas systems.

The only RAMP family that could not be confidently assigned to any of the three groups is Csf3: despite some weak sequence similarity to both Cas6 and Cas5 in the G-rich loop region, these proteins contain fewer predicted beta-strands than Cas6 or Cas7 and no predicted RRM domain downstream of the G-rich loop; although the latter feature resembles the organization of Cas7, there is otherwise no similarity between these proteins.

The diversity and weak conservation of the sequences and structures of the RAMPs hamper the elucidation of the evolutionary relationships between the three major groups. Structural comparisons seem to suggest a specific affinity between the Cas6 and Cas7 groups [16]. From a different standpoint, the most parsimonious evolutionary scenario might involve an ancestral RAMP with a single enzymatically active RRM domain, resembling Cas7, and a single duplication in the putative common ancestor of the Cas5 and Cas6 groups, with subsequent deterioration or displacement of the C-terminal RRM domains in several Cas5 and Cas6 lineages (Figure 3). Under this scenario, the similarity between Cas7 and Cas6 would reflect ancestral structural features.

#### The characteristic arrangement of RAMPs in CRISPR-Cas operons

Mapping the new classification of RAMPs described in the preceding section onto the operons of the type I and type III CRISPR-Cas systems reveals a common pattern of organization. Most subtypes of the Type I CRISPR-Cas systems encode one RAMP of the Cas5, Cas6 and Cas7 groups each. Operons of type III CRISPR-Cas system are organized similarly except that they typically encode multiple Cas7 group RAMPs. Notably, *cas5 *and a *cas7 *usually form a pair of adjacent genes (Additional File 4). Remarkably, the Cas5 and Cas7 orthologs in two distinct CRSIPR-Cas systems belong to the stable core of the CASCADE complex both in *E. coli *(Type I-E) [7,8] and in *S. solfataricus *(Type I-A) [16]. In the unclassified (U-type) CRISPR-Cas system, operons that contain no *cas5*, a *cas7 (csf2) *gene is located adjacent to the *csf3 *gene suggesting that Csf3 is a truncated derivative of Cas5 (Additional File 4). In the unclassified (Type U) CRISPR-Cas systems that contain no *cas5*, a *cas7 (csf2) *gene is located adjacent to the *csf3 *gene suggesting that in these systems Csf3 could play a role comparable to that of Cas5. (Additional File 4).

#### Enzymatic activities and catalytic sites of the RAMPs

Endoribonculease activity involved in CRISPR transcript processing has been demonstrated for four proteins of the Cas6 group, namely the *E. coli *CasE (Cse3), Cas6 from the archaea *Pyrococcus furiosus *and *S. sulfataricus*, and Cas6f (Csy4) from *Pseudomonas aeruginosa*. All these enzymatically active Cas6 proteins contain a conserved motif centered at the catalytic histidine, and a similar motif is found in many RAMP families of both Cas5 and Cas7 groups, especially from type III CRISPR-Cas systems (Figure 3 and see Additional File 1). In most cases, including Cmr4 (COG1336), Cmr6 (COG1604), Csm3 (COG1337), Csm5 (COG1332), Csm4 (COG1567), and MA1928-like families, this motif is located immediately after the predicted first beta-strand of the RRM domain, similarly to the catalytic histidine of Cas6. Despite the weak sequence similarity between the three groups of RAMPs, the presence of the conserved histidine in many members of each group and in nearly identical positions within the RRM domain suggests that this is an ancestral feature and accordingly the original RAMP most likely was an active endoribonculease.

In addition to the catalytic histidine, the enzymatically active Cas6 protein of *P. furiosus *contains a lysine and a tyrosine residues that are essential for the activity and are thought to comprise the catalytic triad of this enzyme together with the conserved histidine [14]. However, these amino acids are not conserved other than in close relatives of *P. furiosus *Cas6. Although several of the other RAMP families also possess conserved polar or aromatic residues that potentially could contribute to a catalytic triad similar to that of the Cas6 endonucleases (see Additional File 1), the exact architecture of the catalytic site of this RAMPs is currently difficult to predict.

Several RAMPs in each of the three major groups contain a motif with a conserved histidine in the C-terminal portion of the RRM domain. At this time, it remains unclear whether any of the RAMPs that lack the conserved histidine in the N-terminal part but contain other (not homologous to the known catalytic ones) conserved histidines closer to the C-terminus (Figure 3 and Additional File 1) are catalytically active.

Given that the Cas6 group RAMPs are dedicated nucleases for the processing of the CRISPR transcripts (pre-crRNA) that produce the crRNAs and appear to be sufficient for this function [13,14,28], most of the other RAMPs might be involved in non-enzymatic functions in the respective Cascade complexes. However, the possibility remains that some of these RAMPs are involved in crRNA-guided mRNA interference. Indeed, mRNA cleavage has been experimentally demonstrated *in vitro *for the Type III CRISPR-Cas system from *Pyrococcus furiosus *[15]. Furthermore, in some CRISPR-Cas systems, catalytically active RAMPs of the Cas5 or Cas7 groups might substitute for the Cas6 activity. For example, in the type I-C systems that lack *cas6*, the Cas5 family proteins contain a conserved histidine in the C-terminal region of the protein that jointly with other aromatic and charged residues that are conserved in subfamily of RAMPs might contribute to the catalytic site of these proteins (see Additional File 1).

### Gene content similarity between Type I and Type III CRISPR-Cas systems

**Figure 4:**
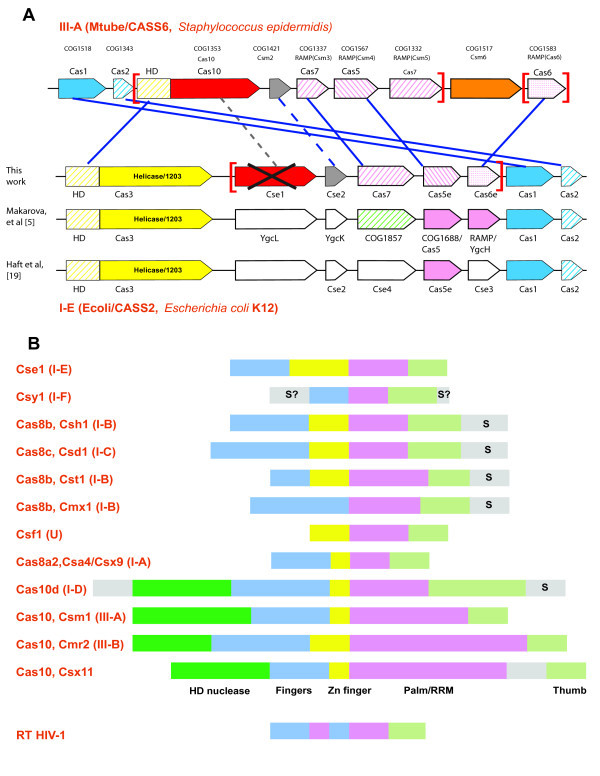
**Gene content similarity between type I-E and type III-A systems and structural organization of large subunits of different CRISPR-Cas systems of type I and III**. A. Genes in the operons for I-E and III-A subtypes are shown by arrows with size roughly proportion to the size of the corresponding gene. Homologous genes are shown by the arrows of same color or hashing. RAMPs are shown by pink or pink hashing. Solid lines connect genes for which homology can be confidently demonstrated, and dashed lines connect genes for which homology is inferred tentatively. The Cascade complex subunits are shown by square brackets. Two previously published domain annotations are included for comparison. B. Domain organization of large subunits of different type I and III CRISPR-Cas systems. Domain size is roughly proportional to correspondent sequence length. The letter "S" marks the regions that could be homologous to small subunits of Cascade complex encoded as separated genes in Type III systems, I-E subtype and some systems of I-A subtype.

The new classification of RAMPs and the common arrangement of RAMP genes in the operons for type I and type III CRISPR-Cas systems emphasize the considerable conservation of organization of the genes encoding (potential) Cascade subunits. The overall organization of the c*as *operon is especially similar between the I-E and III-A subtypes (Figure 4A). Both systems have been experimentally characterized, I-E in *Escherichia coli *[13] and III-A in *Staphylococcus epidermidis *[12,18], and shown to be fully functional. The Type I-E system consists of 9 components, and the Type III-A system includes 10 components (counting the HD superfamily nuclease domains fused to different genes separately). Six genes (domains) of the I-E system, namely *cas1*, *cas2*, *cas3''*(HD), *cas7*, *cas5 *and *cas6e*, are clearly homologous to *cas1*, *cas2*, *cas3''*(HD), *csm3*, *csm4 *and *cas6 *of III-A respectively (III-A contains and additional homolog of *cas7*, *csm5*). Although for the small alpha helical proteins Cse2 and Csm2, sequence similarity cannot be readily detected, they share several similar motifs [5] and might be homologous as well. There are two genes for which there seems to be no counterpart in the other system. One is the Cas3 helicase-nuclease which is unique for Type I systems, and the other is Csm6 which is loosely associated with the CRISPR-Cas systems. The Csm6 protein has been structurally characterized; it contains an HTH domain and probably is a regulatory protein, most likely not involved in the basic CRISPR-Cas mechanism [29].

The large protein Cse1 in the I-E system is a subunit of the Cascade complex [13] and so is the Cas10 protein (CRISPR Polymerase) in *P. furiosus *[15]. Furthermore, Cse1 has a similar size to Cas10 (without the HD domain; Figure 4A). Thus, it seems tempting to speculate that Cse1 might be a homolog of Cas10. As the Cse1 family proteins do not contain any motifs implicated in catalysis in the predicted Cas10 polymerase, Cse1 would be an inactivated enzyme should it be demonstrated that it is indeed a Cas10 homolog.

### Putative homology between the large and small subunits of different type I and type III CRISPR-Cas systems

Among the large subunits of Type I CRISPR-Cas systems, sequence conservation has been demonstrated previously [5] for several subfamilies of the Cas8 family (Cas8a1/Csa6, a subfamily of subtype I-A; Cas8b/Csh1/Cst1, a subfamily of subtype I-B; and Cas8c/Csd1, a subfamily of subtype I-C). Here, using HHpred and PSI-BLAST, we linked several other subfamilies to the Cas8 family: Cmx1/Csx13/LA3191 associated with some diverged variants of I-C subtype and Cas8a2 (Csa4/Csx9 subfamily) associated with some I-A subtype systems. For example, HHpred identifies the profile for Cas8a2 (Csa4) with probability 0.42 using the FRAAL5579 sequence from *Frankia alni *(subfamily Cas8a1/Cst1) as the query and profile TIGR02556 for Cas8a (Cst1 subfamily) with probability 0.83 using the M23134_00692 sequence (Cmx1/Csx13/LA3191 subfamily) from *Microscilla marina *as the query. The large Cascade subunit of subtype I-D shows similarity to the Zn-finger regions of the Cas8b/Cst1of I-B system and additionally is fused to an HD domain analogously to the type III Cas10 proteins. The large subunits of the I-E (Cse1) and I-F (Csy1) subtypes do not show any sequence similarity to one another (despite the fact that these systems are related by the Cas1 phylogeny and the *cas *gene sets) or to any Cas8 family proteins.

Type III systems contain several subfamilies of Cas10 (Csm1, Cmr2 and Csx11 according to [19]) that have been denoted CRISPR polymerases because of their similarity to the Palm/Cyclase domain [2,30,31]. The CRISPR polymerase consists of several domains, namely, the HD domain (predicted nuclease), a distinct domain so far unique to this protein family, a Zn-finger domain, and a region containing the Palm domain, the signature domain of various polymerases and cyclases which adopts a distinct RRM fold [2]. The Palm domain of CRISPR polymerases is more similar to the Palm domain of cyclases than to those of 3'-5' DNA and RNA polymerases, and contains all typical secondary structure elements including four beta-strands of the core RRM fold [31]. Many structures of Palm domain-containing polymerases from all domains of life and numerous viruses have been solved and compared [32]. Most of these polymerases show a common arrangement of core domains and the same modes of nucleic acid binding; the polymerases additionally contain a variety of editing nuclease domains and regulatory domains. The core domains (usually arranged in the same order from the N-terminus to the C-terminus) are the following: the "Fingers" domain that binds a nucleotide, the catalytic "Palm" domain "Palm" that binds single-stranded nucleic acid, and the "Thumb" domain that binds double-stranded nucleic acid [32].

Despite this structural and mechanistic similarity, only the Palm domains of these numerous polymerase families are clearly homologous [32,33]. The most conserved feature of the Palm domains is the beta-hairpin formed by strands 2 and 3 of the RRM fold [33,34]. The thumb domain is usually enriched in alpha helices some of which interact directly with the DNA or RNA duplex [32].

To analyze and compare the sequence and structural features of the large subunits of type I and type III systems (Cas8 and Cas10 [predicted CRISPR polymerases], respectively), we constructed a multiple alignment of representative sequences and predicted the secondary structure using the JPRED program (Figure 4B) (Additional File 5). A detailed analysis of the C-terminal region of CRISPR polymerases (starting immediately after the Zn-binding treble clef domain) showed that a region consisting mostly of alpha-helices follows the fourth strand of the RRM fold of the Palm domain (Region 5 in Additional File 5). This arrangement is consistent with the general structure of Palm-domain polymerases described above and suggests that this region of the CRISPR polymerases could be equivalent to the Thumb domain of other polymerases. Furthermore, given that the core Palm domain is rather compact in these proteins, the region located after the HD nuclease domain and upstream of the Zn-binding domain (Region 2 in Additional File 5) might be an equivalent of the Fingers domain.

Most of the large subunits of different subtypes of Type I CRISPR-Cas systems contain a readily identifiable Zn-finger domain in the middle of the protein sequence [5]. If the large subunits are highly diverged, inactivated Palm-domain containing polymerases as proposed above, and the Zn-finger is equivalent to the treble-clef domain found in the CRISPR polymerase, one should expect that a domain containing several beta-strands compatible with the general structure of the Palm-domain followed by an alpha helical region would be located downstream of the Zn-finger. Indeed, in various subfamilies of Cas8, Cas10d, inactivated Cas10 (Csx11 subfamily) and Cse1, we observed the same structural pattern, namely, at least three predicted beta-strands that could belong to a RRM fold, including the core beta-hairpin, followed by an alpha-helical region (Regions 4 and 5 in Additional File 5). Because two other subfamilies (Csy1 and Cmx1) do not contain Zn-fingers, it is difficult to map the beginning of the putative Palm-domain within these sequences. However, we detected sequence similarity between Cmx1 and Cas8 (see above) and given that Cmx1 proteins possess an alpha-helical C-terminal domain (Regions 4 and 5, Additional File 5), it seems likely that Cmx1 is homologous to Cas8. The Csy1 protein might be homologous to Cse1 (the large subunit of the subtype I-F system) given the overall similarity in the operon organization between the I-E and I-F systems and the clustering of these systems in the Cas1 phylogeny [20]. Like Cse1, Csy1 also has an alpha-helical C-terminal domain and an N-terminal region with mixed alpha-helices and beta-strands (Additional File 5, Csy1 subfamily). Although the pattern of the predicted secondary structure elements of Csy1 cannot be confidently aligned with either Cse1 or Cas8, we cannot rule out the possibility that it contains a derived RRM-like fold. Most of the large subunits of type I CRISPR-Cas systems containing Zn-fingers also possess an N-terminal region with mixed beta-strands and alpha helices which is compatible with the general organization of the region following the HD domain and preceding the Zn-finger in Cas10 subfamilies (Region 2, Additional File 5). Taken together, analysis of the general secondary structure features, the presence of the Zn-finger domain in many large subunits, the similar operon organization and the experimentally demonstrated functional link to RAMPs and the Cascade complex [8,13,15] raise the possibility that all large subunits of CRISPR-Cas systems might be inactivated derivatives of the CRISPR polymerase (Figure 4B). However, there is currently not enough evidence to rule out non-homologous displacement of some of the large subunits or their individual domains.

Interestingly, the pattern of secondary structure elements in the putative Fingers domain in Cas10 and several large subunits, Csx11, Cas8a2/Csa4, Csc3 (Region 2, Additional File 5) resembles the structures of the RRM domain found in RAMPs. Like the RRM core domain, many of the Fingers-like domains contain four predicted beta-strands. Furthermore, the Fingers-like domains start with a beta strand-alpha helix element and ends by a helix-beta-strand element, which are the two most conserved structural patterns in RAMPs (see above and Additional File 1). Thus, it is possible that the Fingers domain of the large subunits adopts an RRM fold.

In several families of the large subunits (Cas8a1, Cas8b, Cas8c, Cmx1 and Cas10d) of the I-A, B, C and D system subtypes, the C-terminal region (predicted Thumb domain) is longer than that in Cas10 proteins (8 alpha helices versus 4 in Cas10;Region 5, Additional File 5). Interestingly, in these subtypes, the small Cascade subunit is missing in the CRISPR-Cas operons. Typically, the small subunit is an alpha-helical protein containing 6 alpha helices (structure is solved for *cmr5*: AF1862, 2OEB and TTHB164, 2ZOP). This is, in principle, compatible with the size of the extra alpha helical region at the C-termini of the aforementioned large subunits (Figure 4B). The Csy1 protein, the subtype I-F specific large subunit, contains eight predicted alpha helices at the C-terminus and four helices at the extreme N-terminus. Because none of the predicted RAMP proteins from this system contain extended alpha-helical regions compatible with the size of the small subunit, we speculate that a domain homologous (or at least structurally and functionally analogous) to the small subunit might be "hidden" in Csy1.

The demonstration that at least some of the large subunits of Type I CRISPR-Cas systems are homologous to the CRISPR polymerase suggests that all these large proteins function and interact with DNA or RNA in a mode analogous to that of other Palm domain polymerases Table 1. In particular, the Palm domain probably interacts with ssDNA whereas the analog of the Thumb interacts with dsDNA. Notably, evolutionarily conserved inactivated derivatives of Palm domain polymerases have been detected in Archaea and eukaryotes although their functions remain uncharacterized [35,36]. The small subunits of CRISPR-Cas systems might be responsible for the recognition of the PAM motif that is required for the selection and incorporation of new spacers.

The conservation of the complete set of catalytic residues typical of Palm domain polymerases and cyclases implies that the Palm domain of Cas10 is enzymatically active but the nature of this activity remains unknown. There is no indication that a processive polymerase is involved at any stage of the CRISPR-Cas system functioning. The possibility remains that Cas10 is a nucleotidyltranferase or even a nucleotide cyclase, perhaps involved in crRNA modification. This is compatible with the activity of the tRNA(His) guanylyltransferase THG1 [37] which belongs to the same clade of Palm domain proteins with Cas10 and the GGDEF diguanylate cyclases [31] (see above). Another possibility is that Cas10 has a secondary role as a helicase in one or more stages of CRISPR/Cas functioning. A helicase activity dependent on the cleavage of the α-β bond in NTP during polymerization has been demonstrated for the bacteriophage T7 RNA polymerase [32,38,39], which is a derivative of the Palm domain DNA polymerases [33]. Remarkably, all Type I CRISPR-Cas systems in which the large subunits are inactivated Cas10 homologs also include the Cas3 helicase, and conversely, all Type III systems that contain Cas10 proteins predicted to be active lack Cas3 [20]. Thus, it is tempting to propose that Cas3 compensates for the loss of the original enzymatic function of Cas10 in Type I CRISPR-Cas system whereas the inactivated derivative of Cas10 performs an accessory structural role. It is of further note that some Type U CRISPR-Cas systems that contain degraded versions of Cas10 and lack Cas3 include a DinG-like helicase (see below), in further support of the possibility that a helicase activity required for the CRISPR-Cas function can be supplied by different, in some cases, unrelated proteins.

#### Type II CRISPR-Cas systems and homologs of Cas9

The signature protein of the type II CRISPR-Cas systems II, Cas9, does not show any detectable similarity to any proteins in Type I and Type III systems. It appears that Cas9 is sufficient both to generate crRNA and to cleave the target DNA [6,9,20]. The large Cas9 protein (~1000 amino acids) contains two predicted nuclease domains, namely, the N-terminal RuvC-like nuclease (RNAse H fold) and the HNH (McrA-like) nuclease domain that is located in the middle of the protein [5,40].

**Figure 5:**
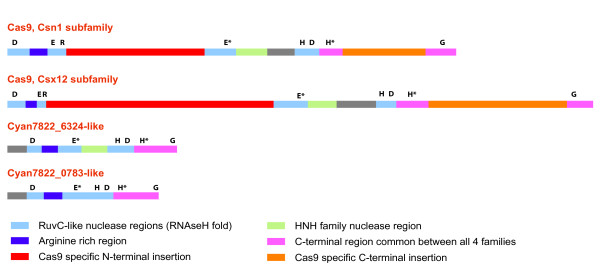
**Structural organization of Cas9 protein families and their homologs**. Homologous regions are shown by the same color. Distinct sequence motifs are denoted by the corresponding conserved amino acid residues above the respective domains (when the same conserved amino acid occurs in different motifs, one is marked by an asterisk to avoid confusion).

To analyze the remaining portions of the Cas9 protein, we constructed a multiple alignment of the two distinct subfamilies of Cas9 (Csn1 and Csx12 subfamilies), predicted the secondary structure and performed PSI-BLAST and HHpred searches with different queries from these subfamilies. Both full-length proteins and fragments outside of previously identified domains were used for these searches (Additional File 6, N-terminal region, N1 and C-terminal region N2). We failed to detect any significant similarity for the region N1 which is located between the two nuclease domains (Additional File 6) and is ~400 aa in length. The predicted secondary structure in this region is mostly alpha-helical with several beta-strands in the middle. For the region N2 which is located downstream of the HNH domain (eg. NMCC_0397 from *Clostridium cellulolyticum *H10, 610 to 1021 aa. Additional File 6), HHpred identifies a weak similarity to the RuvC-like resolvase profile (cd00529; probability 0.22). Given that a region similar to RuvC has been previously detected at the N-terminus of Cas9 [5], we investigated the N2 region in greater detail. Comparative analysis of the conserved motifs and secondary structure of Holliday junction resolvases (HJRs) and endonucleases [40-42] and the regions of similarity with RuvC identified in Cas9 indicates that the N-terminal region contains three known motifs. Furthermore, the region immediately after the HNH-nuclease domain corresponds to the C-terminal region of HJR superfamily which contains two alpha helices (or one long helix) and a fourth motif with the signature HxxD (Figure 5, motifs 1-4 in Additional File 6). Thus, within the RuvC-like domain, Cas9 contains an almost 450 aa long insert which includes the HNH nuclease domain; nevertheless, the RuvC domain is most likely an active nuclease given the conservation of all four HJR motifs and the characteristic conserved secondary structure elements (Additional File 6). For the rest of the N2 region, we failed to detect sequence similarity to any proteins although secondary structure prediction for this region shows that it consists mostly of beta-strands with a few alpha helices, suggesting the presence of a compact globular domain (Additional File 6).

The exact roles of the two predicted nuclease domains of Cas9 remain unclear. However, the insertion of the HNH nuclease domain into the RNAse H fold domain suggests that their activities are closely coupled and that their active sites are proximally located. The HNH nuclease domain, which is common in restriction enzymes and possesses DNA-endonuclease activity [43,44], might be responsible for the target cleavage. Conversely, the RuvC-like RNAseH fold domain might be involved in CRISPR transcript processing.

Several PSI-BLAST searches using various regions of Cas9 as queries detected similarity to a large family of prokaryotic proteins containing both RuvC-like and HNH-nuclease domain (for the details on the identification of these homologs see Additional File 6). This family could be divided into at least two subfamilies by domain architecture (Figure 5). Analysis of the genomic context of the genes encoding these Cas9 homologs did not reveal any stable associations, and there are no CRISPR repeats in the vicinity of any of these genes. Hence, the function of these proteins remains obscure. An intriguing possibility is that they might represent a novel system of RNA-guided DNA interference involved in antivirus defense that in some respects could be analogous to the prokaryotic Argonaute proteins [45]. Some of these proteins form large species-specific paralogous families (e. g. 49 genes in *Ktedonobacter racemifer *or 17 genes in *Microcoleus chthonoplastes*, see Additional File 6). These expansions of closely related paralogs in the same genome suggest that at least this subset of the family could represent novel mobile elements. The *cas9 *gene might have been co-opted by the CRISPR/Cas system from such mobile elements with the concomitant loss of typical CRISPR/Cas components, such as RAMPs and CRISPR polymerases resulting in the emergence of the distinctive Type II gene neighborhoods. The emergence of Cas9 involved two distinct insertions, namely a mostly alpha-helical insert near the middle of the protein sequence and a mostly beta-stranded region near the C-terminus (Figure 5). These large inserts did not show sequence similarity to any other proteins but, given the close functional similarity between Type II and Type I/III CRISPR-Cas, it cannot be ruled out that the inserts originate from CRISPR-Cas components.

#### Type U CRISPR-Cas systems

An unusual CRISPR-Cas system has been recently identified in several bacterial genomes, e.g., *Acidithiobacillus ferrooxidans *ATCC 23270 (operon AFE_1037-AFE_1040) (denoted type U as it did not contain signature genes of any of the three CRISPR-Cas types) [20]. This system is associated neither with the two ubiquitous core *cas *genes, *cas1 *or *cas2*, nor with any other signature genes of the three CRISPR-Cas types or the 10 subtypes. The *A. ferrooxidans *system consists of four genes denoted *csf1*, *csf2*, *csf3 *and *csf4*. The Csf2 protein is a Cas7 group RAMP closely related to the Csm3 subfamily. Csf3 is yet another diverged RAMP protein that might be functionally analogous to the Cas5 group (Figure 3). Csf1 is a Zn-finger containing protein. A PSI-BLAST search started with one of the Csf1 proteins (AFE_1038, *Acidithiobacillus ferrooxidans*) after first iteration identified a weak (not statistically significant) similarity with the Zn-finger sequence of Cas10 proteins of the Crm2 family, and its predicted secondary structure is comptabile with the treble clef fold. The secondary structure prediction for these proteins generally shows the same pattern as in the large Cascade subunits discussed above, namely several beta-strands (some of them forming a potential hairpin) and several alpha-helices at the C-terminus (Additional File 5). Taken together, these observations suggest the possibility that Csf1 could be a highly divergent, inactivated and N-terminally truncated Cas10-like polymerase derivative lacking the N-terminal Fingers domain. The fourth gene in this system, *csf4*, is usually located on the complementary DNA strand in the divergent orientation and encodes a DinG family helicase [46]. According to the CRISPRdb database [47], CRISPR arrays are present in the vicinity of the above four genes in all of the respective genomes but the architecture of these arrays is unique in each case. Thus, this system might function in conjunction with different CRISPR arrays and would not require a distinct repeat signature.

Homologs of Csf1, Csf2 and Csf3 were identified in several Actinobacteria in a somewhat different genomic context (eg. pREL1_0084-pREL1_0087 *Rhodococcus erythropolis*). There is no DinG-like helicase in the neighborhood. A gene encoding a small, largely alpha-helical protein with conserved positively charged and aromatic amino acids in several positions follows the *csf1 *gene. This arrangement resembles the large and small Cascade subunits of the I-E and III-A subtypes. All these loci are located on plasmids. There are no CRISPR repeats detected on these plasmids and, in many cases, in other partitions of the respective genomes either (see the CRISPRdb database [47]). Thus, this variant of the Type U CRISPR-Cas system might be a mobile Cascade-like module functioning in a completely different context, not associated with CRISPR repeats and other Cas proteins.

#### Unusual CRISPR-Cas system variants

**Figure 6:**
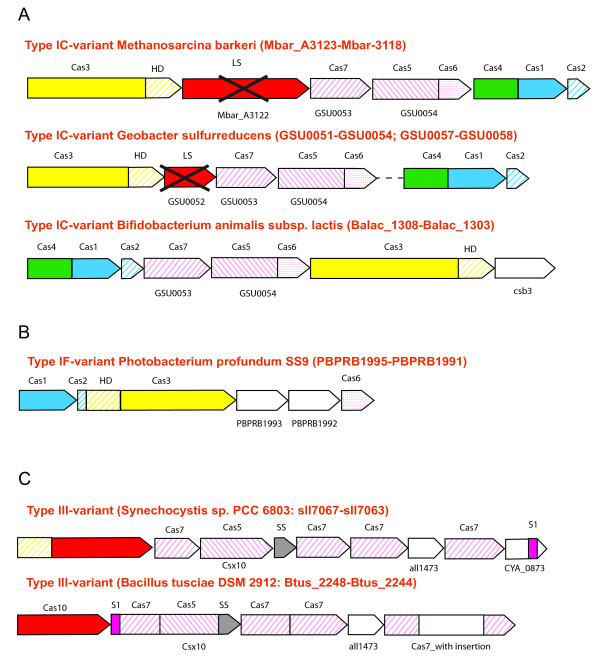
**Unusual CRISPR-Cas systems**. **A**. Type I-C-variants with GSU0054 (or GSU0053) signature gene. **B**. Type I-F-variant. **C**. Type III-variant.

A few CRISPR-Cas systems that could be readily classified into established subtypes or at least types based on signature genes contain, in addition, unusual protein families, domain fusions and/or operon rearrangements (Figure 6). For example, a distinct subtype I-C system variant has a number of specific features, in particular, fused *cas1 *and *cas4 *genes and two extremely divergent RAMPs (Figure 6A). One of the latter is a homolog of Cas7 group RAMPs (GSU0053), and the other one is an apparent fusion of Cas5 and Cas6 group RAMPS (GSU0054) (see Additional File 1). The ancestral version of this systems could be similar to that present in *Methanosarcina barkeri*, with a probable homolog of Cas8 (inferred Cas8 family protein with characteristic alpha-helical domain at C-terminus which could also include fusion to the small subunit). Several CRISPR-Cas systems (e.g. in *Geobacter sulfurreducens*) contain an apparent deteriorated version of the Cas8 protein (which is identified on the basis of presence of alpha-helical C-terminal domain and the location in the operon). In a few other genomes there are no traces of a Cas8-like subunit (e.g. in *Bifidobacterium animalis*). The additional gene in this operon (Csb3 family) resembles RAMPs of the Cas6 family by secondary structure prediction and several motifs (see additional file Additional file 1); however, this protein also contains a C-terminal extension resembling the alpha-helical region present in Cas8 family proteins. The variant of the subtype I-F system in *Photobacterium profundum *contains three genes that are clearly orthologous to Cas1, Cas2/Cas3 fusion and Cas6f of the I-F system, respectively; however, two additional genes in this system encode proteins (PBPRB1993 and PBPRB1992) that show no detectable sequence similarity to any known protein families (Figure 6B). By length and the position in the operon, these proteins resemble Csy2 and Csy3, respectively. The predicted secondary structures of these proteins are also compatible with the RAMP structure but not with that of the Cas8 family (no alpha-helical domain). Thus, these proteins might belong to the Cas5 and Cas7 groups, respectively. The *cas8 *(large subunit) gene is absent in this system, which seems active based on the presence of large array of CRISPR repeats in the genome.

Some variants of the subtype III-B system encompass the signature Csx10 family which belongs to the Cas5 group of RAMPs (Figure 6C). Another feature of this system is the presence of a protein of all1473 family, which does not show any similarity to known Cas protein families but the predicted secondary structure resembles that of the RAMPs. These systems also contain the ribosomal protein S1 domain (the OB fold [48] which forms two distinct fusions). In some systems (e.g. in *Bacillus tusciae*), several additional fusions occurred, mostly between adjacent genes in the operon (Figure 6C). The Cas10 homolog in the latter systems lost the HD domain but retained all catalytic residues of the Palm domain.

Comparative analysis of these unusual variants of CRISPR-Cas system architectures may shed additional light on CRISPR-Cas evolution as discussed in the next section.

#### An evolutionary scenario for the origin of CRISPR-Cas systems

**Figure 7:**
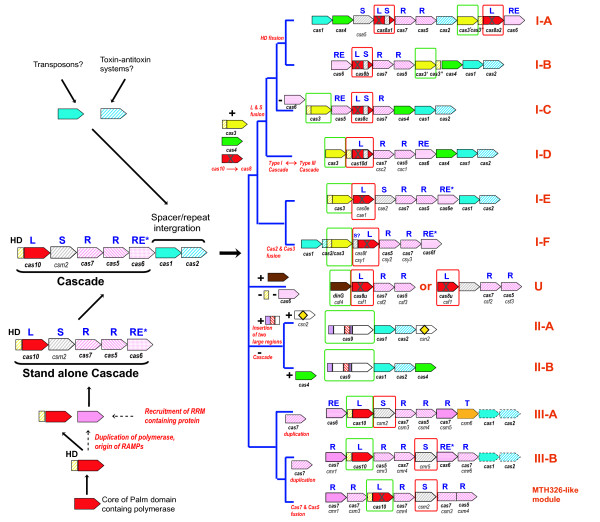
**Evolutionary scenario for the origin of CRISPR-Cas systems**. Homologous genes are color-coded and identified by a family name (names follow the classification from [20]). Names in bold are proposed systematic names including those propose in this work; "legacy names" are in regular font. The signature genes for CRISPR-Cas types are shown within green boxes, and for subtypes within red boxes. The bold letters above the genes show major categories of Cas proteins: L, large CASCADE subunit; S, small CASCADE subunit; R, RAMP CASCADE subunit; RE, RAMP family RNase involved in crRNA processing (experimentally characterized nucleases shown be asterisks); T, transcriptional regulator. Genes coding for inactivated (putative) polymerases are indicated by crosses. Major evolutionary events are shown in the corresponding branches. Broken lines denote alternative evolutionary scenarios for the origin of RAMPs.

Combined, the findings described here allow us to propose a simple scenario for the origin of the CRISPR-Cas system (Figure 7). The primary observations that contribute to this reconstruction of CRISPR-Cas evolution are:

i) the demonstration that Cas7 proteins represent a distinct group of RAMPs

ii) classification of all RAMPs into three major groups, Cas5, Cas6 and Cas7

iii) the more tentative unification of Cas8 and Cas10 into the CRISPR polymerase family (large subunits of CRISPR-Cas systems)

vi) the tentative unification of small, Csm2-like subunits

Taking into account these newly discovered unifying connections between the Cas proteins, comparison of the gene composition and operon organization of the three major types and 12 subtypes of CRISPR-Cas systems allows us to reconstruct the ancestral forms with confidence.

The ancestral functional CRISPR-Cas system probably resembled Subtype III-A and consisted of six or seven genes, namely the two universal *cas *genes, *cas1 *and *cas2 *("information processing" subsystem involved in the adaptation phase) along with four or five additional genes which comprised the "executive" subsystem (CASCADE complex) involved in crRNA processing and interference. The "executive" module included the large subunit (Cas10/Cas8, or the CRISPR polymerase), the small subunit (an alpha-helical protein or domain enriched in positively charged and aromatic amino acids) and two or three RAMPs (of the Cas5, Cas6 and Cas7 groups). Given that Cas5 and Cas6 are structurally similar and considering that Cas5 probably substitutes for Cas6 in subtype I-C, the ancestral system could have contained only one protein representing these two families. Most of the ancestral components are retained in many extant CRISPR-Cas subtypes, in particular, the Type III systems that show relatively little variation. In the most parsimonious scenario, relatively few evolutionary events are required to explain the emergence of Type I and Type III systems with their subtypes (Figure 7)

The key events that gave rise to Type I CRISPR-Cas systems events include the acquisition of the helicase Cas3 and the RecB family nuclease Cas4; inactivation of the Palm domain of Cas10 protein that yielded Cas8; and fission of HD domain and Cas10 followed by fusion of HD domain with the Cas3-like helicase. The preservation of 6-7 ancestral components in most of the Type I and Type III CRISPR-Cas systems suggests tight structural and functional links among these proteins. However, a degree of independence between the "informational" and "executive" modules has been reported previously [5,19,20]. In particular, Type III "executive" modules (type III Cascades) are often encoded separately (not in proximity to *cas1 *and *cas2 *genes) and often occur in a genome along with Type I and/or Type II systems. Furthermore, Cas1 sequences from Type III systems are not monophyletic in the phylogenetic tree [20], suggesting that Type III "executive" modules have combined with diverse "informational modules" on multiple occasions. This is a likely evolutionary scenario for Subtype I-D in which the Cascade complex (especially the Cas7 group RAMP Csc2) resembles the Type III counterpart rather than other Type I Cascades (See Additional file 1). Interestingly, HD domain in this subtype is associated with the large subunit (Cas10d) rather than with Cas3, again similarly to Type III rather than to other Type I systems. However, the HD domain of Subtype I-D systems does not show the circular permutation that is characteristic of the HD domain fused with Cas10 in Type III systems. Thus, in this case, the similarity of domain architectures seems to be convergent, i.e., the HD domain in Subtype I-D systems probably was translocated from *cas3 *to inactivated *cas10 *(or fused with the latter if the ancestral form was a stand-alone HD domain).

There are currently no archaeal or bacterial genomes that would possess the "information processing" module but not the "executive" module of the CRISPR-Cas system. Although involvement of Cas1 in various repair processes has been suggested by recent experiments [49], this tight linkage indicates that the primary function of Cas1-Cas2 depends on the Cascade complex (the "executive module"). In contrast, "Cascade only" systems (Type-U) that are not associated with CRISPR arrays have been identified, suggesting the intriguing possibility that some variants of Cascade might function as an independent defense system, without relying on Cas1, Cas2 and CRISPR arrays for the acquisition of spacers. Although the source of RNA guides for such a system is unclear, an interesting possibility is that this version of Cascade might recognize alien DNA molecules and process nascent alien mRNA to generate RNA guides; such mechanism obviously would be analogous to the siRNA branch of the eukaryotic RNA interference systems [50]. From the evolutionary perspective, such standalone Cascades could be one of the antecedents of CRISPR-Cas systems.

The ancestor of the CRISPR polymerase (Cas10) could have originated from an ancient Palm domain polymerase, such as reverse transcriptase. On the basis of a number of derived shared characters, the CRISPR polymerase has been classified as a member of a distinct group of Palm domain proteins that also includes Thg1-type 3'- 5' nucleic acid polymerases and adenylate and diguanylate cyclases [31]. The association with the HD domain probably goes deep into the evolutionary past given that HD family hydrolases are also commonly associated with the GGDEF family diguanylate cyclases [31,51]. The ancestral function of the CRISPR polymerase that was probably associated with the HD hydrolase domain could potentially involve a distinct form of signal transduction, a role in repair and/or in antivirus defense. The latter possibility seems attractive given the tight association of this protein with the CRISPR-Cas systems.

Genomic islands, in which viral defense, mobile elements and stress response genes, such as toxin-antitoxin systems, are often present together, are likely to be "melting pots" for the emergence of new functional systems through recombination, duplication and lateral transfer [45,52]. It appears likely that the CRISPR-Cas systems evolved in such genomic environments, in part by combination of distinct mobile elements. The origin of RAMPs remains an enigma: these highly diverged RRM-domain proteins possess shared derived characters that are strongly suggestive of their monophyly (such as the presence of a glycine-rich loop and a conserved histidine implicated in catalysis in numerous RAMPs) but do not show significant similarity to any other proteins. An intriguing possibility is that there is a direct evolutionary connection between the CRISPR polymerase and the RAMPs given that the cores of all these proteins consist of RRM domains. The first RAMP proteins could have emerged by duplication of an inactivated polymerase followed by rapid evolution that involved the emergence of the endoribonuclease catalytic center. The ancestral RAMP might have resembled Cas7 proteins that contain a single RRM domain with structural embellishments along with (in some of the Cas7 proteins) a Zn-finger domain, and so resemble polymerases in their domain architecture. Furthermore, several CRISPR-Cas systems apparently remain functional despite having a highly degraded form of the large subunit (type U system) or lacking the large subunit altogether in some variants of Subtype I-C and Subtype I-F (Figure 6B), suggesting that RAMPs could substitute for the function of large subunits. The Cas6 and Cas5 group RAMPs could have subsequently evolved from the Cas7-like RAMPs. This scenario seems plausible considering that RAMP duplications, including tandem duplications and fusions, are often present in CRISPR-Cas loci, especially among the Type III systems in which Cas7 group RAMPs are particularly prone to duplication. Interestingly, in both Type I Cascade complexes that have been characterized in detail, those from *E. coli *and *S. solfataricus *[8,16] the Cas7 subunit is present in multiple copies. It seems plausible that in Type III Cascades, these homo-oligomers are replaced by hetero-oligomers made of paralogous Cas7 proteins. Furthermore, recent inactivation of the CRISPR polymerase (Cas10) was detected in some Type III systems such as MTH326-like (Figure 7). All these observations attest to the dynamic character of the evolution of CRISPR-Cas systems and might add to the plausibility of the route of evolution from the CRISPR polymerase to the RAMP-based Cascade complexes (Figure 7). However, this scenario remains speculative given the absence of specific similarity between the RAMPs and CRISPR polymerases, and recruitment of another RRM-domain protein as the ancestral RAMP gene cannot be ruled out.

The CRISPR polymerase and the entire ancestral, Subtype III-A-like CRISPR-Cas system most likely evolved in thermophilic Archaea. Indeed, this system and in particular the c*as10 *gene is present in a substantial majority of archaea and is confidently reconstructed as a gene present in Last Archaeal Common Ancestor (LACA) [53]. By contrast, Type III CRISPR-Cas systems are much less common in bacteria and often contain variants of Cas10 that are predicted to be inactivated [20]. Like most antiviral defense systems, CRISPR-Cas is prone to HGT and could have rapidly spread among bacteria. Notably, many thermophilic bacteria possess Type III systems, which might have started the dissemination of CRISPR-Cas among bacteria. The active Cas10 could be particularly beneficial in thermal environments, in agreement with the previous observations that identified *Cas10 *as a prominent genomic determinant of the thermophilic life style [2,54].

The close association between Cas1 and Cas2 is more difficult to explain in terms of function or evolution. Given that Cas1 is a DNAse with a Holliday junction resolvase-like activity [21,49], it is most likely to function as a recombinase and integrase at the spacer acquisition stage. These activities are typical of transposable elements, so the origin of Cas1 from this type of elements that are extremely common in prokaryotes appears likely. The endoribonuclease Cas2 might have evolved from another class of equally widespread mobile elements, namely toxin-antitoxin systems. Cas2 is yet another RRM-domain protein that is homologous to VapDHi, the toxin of the two-component toxin-antitoxin system vapDHi/VapX [55], as suggested previously [5] and supported by new HHPred searches which unequivocally retrieved Cas2 as the protein family most similar to VapDHi (for example, a HHpred search started with Psta_3906, VapDHi from *Pirellula staleyi*, detected Cas2, PF09827, as the best hit with the probability 98.9). It remains unclear whether Cas1 and Cas2 ever formed a distinct two gene unit or have independently joined the evolving CRISPR-Cas system.

Type II CRISPR-Cas systems are the only group for which the origin of Cascade complex components could not be confidently inferred. Nevertheless, experimental data suggests that it functions in general similarly to the Cascade complexes of Type I and Type III systems [9]. Of the three types of CRISPR-Cas systems, the Type II systems have undergone the most radical transformation compared to the inferred ancestral form during which the genes encoding the subunits of the ancestral Cascade complex as well as the large (polymerase) and small subunits appear to have been replaced by a single large, multidomain protein, Cas9 which contains two unrelated nuclease domains (Figure 5) and appears to be responsible for both the CRISPR transcript processing and interference.

## Conclusions

The CRISPR-Cas systems are extremely variable in their gene composition, and most of the *cas *genes evolve fast compared to other genes in prokaryotes. Accordingly, the comparative analyses of the Cas protein sequences and structures present a history of progressive detection of increasingly subtle relationship leading to unification of protein families previously thought to be unrelated ([2,5,19,20]. and see Figure 4). The observations described here take this unification a step further. In particular, we substantially expanded the class of RAMPs and showed that at a high level the Cas proteins can be classified into no more than a dozen major groups of families including the Cas1-Cas10 proteins, another group of small subunits (Cas11?) and additionally a few regulatory protein families such as *csm6*. The majority of the families that have been left with historical "legacy" names in the recently published CRISPR-Cas classification scheme [20] now can be assigned to well-defined, "numbered" groups of *cas *genes (see Additional File 7). The results of this analysis emphasize that the CRISPR-Cas systems are built around RRM domains that reach extreme diversification in the RAMPs. This diversity along with recombination between different CRISPR-Cas loci makes more detailed classification and functional prediction for the CRISPR-Cas systems in the rapidly growing collection of archaeal and bacterial genomes a difficult challenge.

The unification of numerous Cas proteins into the three major groups of RAMPs and the more tentative demonstration of the probable origin of large subunits of diverse CRISPR-Cas systems from CRISPR polymerases together suggest a simple scenario for the origin and evolution of the CRISPR-Cas machinery in thermophilic archaea. Under this scenario, the CRISPR-Cas systems started from a large protein that combined the polymerase and HD hydrolase domain and might have functioned as a stand-alone antivirus defense system. The next step of evolution might have involved duplication of the RRM portion of the polymerase followed by inactivation that produced the ancestral, Cas7-like RAMP or a recruitment of a distinct RRM-domain protein that became the ancestral RAMP. Regardless of the origin of the ancestral RAMP genes, it has undergone a series of additional duplications and rapid diversification that yielded the stand-alone Cascade complex. The formation of the ancestral CRISPR-Cas system was then completed through the unification of Cascade with Cas1 and Cas2. The central theme of this scenario is the origin of the components of the CRISPR-Cas system from different classes of mobile elements. Other prokaryotic defense systems such as restriction-modification [56,57] and toxin-antitoxin systems [58,59] also comprise of such elements, indicating a major trend in the relationships between prokaryotes, viruses that infect them, other classes of selfish element and defense mechanisms.

## Methods

The *cas *gene nomenclature follows the recently published CRISPR-Cas classification [20]. Protein sequence searches were performed using PSI-BLAST [60] with an inclusion threshold E-value of 0.01 and no composition-based statistical correction The NR (non-redundant) database (the default for PSI-BLAST searches at NCBI) was used for all searches unless indicated otherwise. In addition, distant similarity detection approaches were applied, namely RPS-BLAST with default parameters to search the conserved domain database (CDD) search [61] and the HHpred search that is based on the comparison of protein family profiles using the Hidden Markov Model technique [27,62]. For the HHpred searches, single sequences were used as a queries, and the SCOP, PDB, PFAM, CDD, TIGRFAM and COG databases were searched as represented on the HHpred server, the default program parameters. Following the recommendations of the HHpred authors, we used the reported probability of a true positive match rather than the e-value to assess the statistical significance of a hit [27]. Multiple alignments of protein sequences were constructed by using the MUSCLE program [63], followed by a minimal manual correction on the basis of local alignments obtained using PSI-BLAST [60] and HHpred [62]. Protein secondary structure was predicted using the Jpred program [64] and these results were used to improve alignment between families within superfamily. Structural comparisons were performed using the DALI server [65].

## Competing interests

The authors declare that they have no competing interests.

## Authors' contributions

KSM and EVK designed the study; YIW wrote the custom scripts for data analysis; KSM and LA obtained the data; KSM, LA and EVK performed data analysis; KSM, LA, YIW and EVK wrote the manuscript. All authors read and approved the final manuscript.

## Reviewers' comments

### Reviewer's report 1

Prof. Malcolm White (nominated by Dr. Purificacion Lopez-Garcia), University of St Andrews, St Andrews, Fife, UK

#### Main comments

The CRISPR system has a fundamental complexity, probably due to rapid evolution and lateral gene transfer, that can be bewildering to those not deeply steeped in the field. Recent crystal structures (notably Cas6 and Cas7) have clarified the relationships of CAS proteins somewhat, and the authors here aim to augment this with extensive bioinformatic analyses to begin a unification (and hopefully simplification) of the plethora of CAS proteins that present a significant barrier to understanding. Finally, one possible evolutionary scenario is outlined. This is an interesting and provocative paper. Many predictions are made, some on shakier grounds than others. The manuscript synthesises a vast amount of bioinformatic analysis and will be a valuable resource for anyone interested in the CRISPR system - whether or not they agree with many of the predictions made in it.

#### Specific comments

1) Firstly, I wonder if the term "RAMP" has lost utility and should be cast on to the bonfire of nomenclature? We now know that RAMP proteins are essentially CAS proteins with an RRM fold - is there any need for another term? They are not even particularly mysterious any more. On the other hand, the RRM fold puts Cas proteins firmly in a functional category.

Response: *In our view, there is still utility in the name RAMP. It was introduced by us back in 2002 (ref. 2) and originally meant "Repair associated mysterious proteins". Later Haft at al. creatively renamed them "Repeat associated mysterious proteins" *[19] *when it became clear that these proteins are part of the CRISPR-Cas system. It makes sense to have a special name or acronym for these RRM-containing proteins because they appear to constitute a monophyletic superfamily. There are occasions when putative RAMPs seem not to be associated with a CRISPR-Cas system - at least they do not belong to cas operons, for example, COG1851 described in ref. 5. Thus, replacing RAMP with something like CARRM (CRISPR-associated RRMs) might be inaccurate or at least premature. It is true that 'mysterious' in RAMP might not be particularly relevant anymore (a few mysteries remain but probably will be resolved soon) but we believe that for the time being this historically rooted acronym is better kept*.

2) The statement that the Pyrococcus CRISPR-Cas system targets RNA (Page 5 line 84) is misleading. Pyrococcus almost certainly has a CASCADE-like DNA targeting system too. Please clarify the text.

Response: *Yes, we agree and have clarified the respective part of the text*.

3) Following on from my first suggestion, to my mind it would be clearer to say that Cas7 shares an RRM domain with many other CAS proteins, rather than classify it as a RAMP (line 130). After all, RNA recognition is probably what many, or all, of the RAMP proteins are actually doing.

Response: *On this point, we do not agree. As indicated above, the RAMPs form a well defined superfamily, and Cas7 is a family within this superfamily (as we show in *Figure 1*). Moreover, the part of the manuscript concerning RAMP classification explains in detail the reasons behind combining several subfamilies into the Cas7 family within the RAMP superfamily. It is indeed very likely that the general function of RAMPs is RNA recognition and binding as first proposed in our 2006 paper *[5]. *However, this general functional description does cover the subject because there certainly is functional specialization among the RAMPs - for instance, some families are catalytically active whereas others are not*.

4) The sentence "Structural comparisons seem to suggest an affinity between the Cas6 and Cas7 groups" (line 218) is correct - they share an RRM domain as stated earlier in this paper. Need this be repeated?

Response: *This is not only about the shared RRM domain but rather about the possible specific affinity between Cas6 and Cas7 as we clarify in the revised text. So this is not a pointless repetition of a previously made statement, and has to stay in a form modified for clarity and emphasis*.

5) The paragraph beginning line 226 notes that cas5 and cas7 genes are usually adjacent. It would be appropriate here to report that the proteins are known to form the core of the Type1 CASCADE complexes both in E. coli (several REFS) and S. solfataricus (REF 16).

Response: *Yes, we agree; a sentence to that effect and references are added:" *Remarkably, the Cas5 and Cas7 orthologs in two distinct CRSIPR-Cas systems belong to a stable core of the CASCADE complex both in *E.coli *(Type I-E) [7,8] and in *S. solfataricus *(Type I-A) [16]."

6) The paragraph beginning line 238 deals with a presumed conserved catalytic histidine identified in Cas6 and also claimed to be present in many representatives of Cas5 and Cas7. I take strong issue with this claim. The histidine is in fact very poorly conserved, even within the Cas6 nucleases - I challenge the authors to identify a candidate in the equivalent position in crenarchaeal Cas6's for example. Likewise, I see no conserved histidine in the Cmr4 and Cmr6 proteins that I am most familiar with. Indeed it would be unusual if a catalytic residue was observed in these examples, as they are almost certainly not catalytic subunits (eg *E. coli *CASCADE has no nucleolytic activity). I think this needs revision, along with the accompanying figure where the blue line represents the "conserved" active site.

Response: *It is true that presumed catalytic histidine is not 100% conserved even in the proteins of the Cas6 group, and representatives of the Cmr4, Cmr6 and Cas5 families indeed exist that lack this histidine. However, we presented alignments of all RAMP families (for these alignments, we tried to select the most diverse representatives for each family) discussed in this work in *Additional File 1 *to back all our claims. The general conservation of the histidine in Cmr4 is strong (100% for our set of 30 selected diverse sequences), and the same holds for the Csm4 and Csb3 families; in other families (e.g. Cmr6) the conservation is less pronounced but still traceable in at least 50% of the sequences (see the consensus shown in the *Additional File 1*). Given that these histidines are located after the first beta-strand of the (first) RRM domain, they are most likely to be homologous. Neither Cas7 nor Cas5 families contain this particular partially conserved histidine, which is consistent with the absence of nucleolytic activity of CASCADE in E.coli. However, as we pointed out in the text, in the type I-C systems, the Cas5 family probably substitutes for the Cas6 function using a potential catalytic histidine in a different location. Certainly, for some of the families that contain the partially conserved histidine, the catalytic activity is only a prediction. We cannot rule out that in some families this histidine performs a structural role, and that these proteins are not active nucleases. Conversely, we cannot confidently claim that the families that lack the conserved histidine are not active nucleases: in the case of different Cas6 subfamilies, we already know that different amino acids can contribute to the catalytic activity. Taking into account all these and additional considerations in the text, considerations, we believe that it is appropriate to keep the original version of *Figure 3, *especially given that the main purpose of this Figure is to show a hypothetical scenario of RAMP evolution. However, to clarify the hypothetical character of our predictions of nuclease activity in RAMPs other than the Cas6 group, we added words of caution throughout the respective parts of the text and the legend to *Figure 3 .

7) From line 319 onwards, the authors refer to a "zinc finger" domain in Cas10. A zinc finger is a very narrowly defined subset of the much larger group of zinc binding domains. Is this really a zinc finger? If not then a global replace with "zinc binding domain" would be appropriate.

Response: *Zn-finger is appropriate here. Zn-fingers actually represent a structurally diverse class of domains that chelate one or more Zn ions (e.g. various Treble Clef domains, C2H2-like, WRKY and BED domains that have ostensibly different folds)*. 

The speculation that Cse1 and Cas10 are homologous, in the absence of any bioinformatic evidence other than that they are "a similar size" seems unwarranted, particularly as the two proteins seem to have very different properties based on the available biochemical data.

Response: *We are not aware of biochemical data showing that these proteins possess very different properties. The only piece of evidence known to us is that these proteins are subunits of the CASCADE-like complexes (CASCADEs in E. coli *[13] *and Pseudomonas *[66], *and the Cmr complex in Pyrococcus *[15]). *This information seems to be compatible with the speculation that these proteins are highly diverged homologs although we certainly admit and indicate in the article that the indications are weak*.

The authors return to this theme (line 352) and here provide some data based on secondary structure prediction. These paragraphs could be merged. However, the data linking Cas10 with Cse1 and Cas8 is really very weak and there is a good chance that they are not related. When a possible evolutionary relationship between two proteins seems to depend on the prediction of shared "mixed beta-strands and alpha helices" (line 373), it is hanging by a very thin thread indeed. To be fair, this is explicitly acknowledged later (line 380), but then the authors go on to assert that "at least some of the large subunits of Type 1 systems are homologous to the CRISPR polymerase", and then suggest commonalities in RNA and DNA binding. For me, the relationship is not at all proven from the available biochemical and bioinformatic evidence.

Response: *The homologous relationship between Cas10 and large subunits of type I systems is indeed a hypothesis (one may choose to use 'speculation' instead); hopefully, the structures of these proteins will be solved soon enough - this should settle the issue. However, combining all the available data and all the indirect bioinformatic evidence, we would rather submit that there is a low chance that they are not homologous. Here we summarize all the lines of evidence once again:*

1. Both Cas10 and the Type I large subunits (LS) are parts of CRISPR-Cas systems

*2. They are encoded in very similar contexts in the respective operons (see the *Figure 1A *and *7*)*

*3. These proteins are similar in size (Cas10 is compared without HD domain; see *Figures 1A *and *1B)

4. Cas10 and LS form CASCADE-like complexes with RAMPs

5. Many LS contain Zn fingers in the middle of the protein - similarly to Cas10

*6. Secondary structure analysis suggests the same general architectures of Cas10 and LS, which is also similar to the general organization of Palm domain-containing polymerases*.

7. Secondary structure of the region following the Zn-fingers (where present) for most LS is compatible with the RRM fold

*For typical predictions based on the 'guilt by association' approach, the first four points would be sufficient to predict the analogous functions for these subunits (such a functional link between LS and Cas10 has been suggested in our 2006 paper *[5]). *Homology of these proteins appears to be the most parsimonious (the simplest) explanation for these observations. The last three points reinforce this hypothesis stronger by decreasing the likelihood that all these common features evolved independently. There are also numerous additional issues such as the inactivation of the predicted catalytic aspartates in the Palm domains and the lack of the HD domain in some Cas10 proteins, the dramatic divergence in the adjacent genes in the respective operons, and indications of domain loss and deterioration of the LS leading to the rapid loss of sequence similarity that could be brought into the discussion. More generally, we should note that rapid divergence of many components of the CRISPR-Cas systems including most of the RAMPs certainly is their salient and important feature. The biological connotations of this rapid evolution remain to be characterized. All of this notwithstanding, in the text of the paper, we point out that non-orthologous displacement of some of the LS is a distinct possibility. We believe that in this discussion, taken together with the reviewer's points, all relevant issues are covered*.

9) The function of the "polymerase" domain of Cas10 is certainly open to question. The suggestion that this domain may function as a helicase and might even act as a functional equivalent of Cas3 (paragraph beginning line 412) is ingenious although I suspect incorrect. It e would be interesting to ascertain whether the activity of the Pyrococcus CMR complex requires ATP hydrolysis in vitro - this is not really addressed in the paper published in 2009.

Response: *We indeed have little hard data to make any strong prediction on the function of Cas10. Back in 2002, we believed that it was a polymerase and presented several considerations in support of this hypothesis that now seems unlikely to be true considering all we know about the CRISPR-Cas systems. Thus, we seem to be essentially left with a cyclase activity but this is again hard to reconcile with the available experimental data. The speculation that Cas10 is a functional equivalent of Cas3 is at least consistent with the observation that the (predicted) active Cas10 is present mostly in the CRISPR-Cas systems lacking Cas3*.

10) Finally, the scheme in Figure 6 outlining a possible scenario for the evolution of the CRISPR/Cas system is by its nature speculative of course. On the one hand, the identification of the RRM domain as a ubiquitous component of CRISPR is very welcome and the sub-division of this classification into Cas5, Cas6 and Cas7 is interesting. I would perhaps include Cas10 as the fourth type of RRM containing protein. As I have stated above, I am not convinced that the "large subunits" - Cas10, Cse1, Cas8 etc, are all homologous, though I concede that they may turn out to be - structures will be required. It is not clear to me why evolution of the system should have started with Cas10 - seems equally likely that this is a quite specialized derivative of the system and that a progenitor might have had a ramp protein plus an HD domain as a simple viral defense unit

Response: *As repeatedly pointed out in this article and in previous publications, regardless of its specific function, Cas10 is clearly homologous to Palm domain polymerase, and its secondary structure is compatible with the overall structure of those polymerases (Fingers, Palm and Thumb domains). Beyond doubt, the Palm domain polymerases comprise an ancient protein family antedating modern cellular life. Therefore it seems highly plausible that the evolution of the CRISPR-Cas system per se started from the polymerase-like protein. Furthermore, fusion of polymerases (the HD domain in the case of CRISPR-Cas) with nucleases is a pervasive theme in the evolution of replication and repair systems *[2,67]. *Thus, the polymerase and the polymerase-nuclease fusion chart a plausible path of evolution from the general replication machinery to the CRISPR-Cas system. In a sharp contrast with Palm domain polymerases, the RAMPs are highly specialized and more or less restricted to CRISPR-Cas systems which makes them unlikely ancestors. We certainly do not deny that the evolutionary scenario presented is speculative but we believe there is a strong logical underpinning behind it*.

#### Minor points

Table 1. The prediction of DNA nuclease activity for Cas10 seems to discount the available biochemical evidence for the role of Cmr2.

Response: *We are not sure what the relevant data is. The prediction of nuclease activity for the HD domain is confident*.

Line 104 - "and" missing

Response: we checked and double checked this line and the lines around it but could not identify a place to insert "and".

Line 120 - "functioning" should be replaced by "function"

Response: *Replaced*.

### Reviewer's report 2

Dr. Frank Eisenhaber, Bioinformatics Institute (BII), Agency for Science, Technology and Research (A *STAR), Singapore

This work describes an exhaustive sequence analysis of the protein components of the CRISPR-Cas modules in the framework of the sequence homology concept. The strength of this article is the finally derived, highly plausible evolutionary scenario of the protein modules that is of striking simplicity. This is a very strong biological argument supporting the conclusion chain of this work.

The latter helps to moderate the awkward impression from sometimes very lousy significances of alignments that would not be worth being mentioned outside of this context (the worst case being 0.42 for the match of the profiles of Cas8a2 and Cas8a1, msp 15).

*Response: Obviously, we would never propose that these families are homologous on the basis of "lousy" HHpred probability value. The hypothesis makes sense only within the context of the entire analysis and considerations presented in this paper which include genomic context, domain composition and secondary structure compatibility evidence. The reviewer concurs that collectively this amounts to evidence worth consideration*.

It is not good to present such data as strong findings; some self-critical, moderating comments with regard to such cases would enhance the article.

*Response: We have never claimed that these were strong findings. There were quite a few moderating comments even in the original manuscript although we are not sure in what sense such comments are supposed to be self-critical as long as nothing is misrepresented. The revision includes additional words of caution as well as further explanations, in particular, in the responses to Reviewer 1*.

#### Minor points

1) Reference style in Figure 4 does not fit the style of the reference list. Please add the reference number.

*Corrected*.

2) Msp 5 (line 91) has an obvious typo ("revealed = distant").

*Corrected*.

### Reviewer's report 3

Dr. Igor B. Zhulin, Department of Microbiology, University of Tennessee, Knoxville, TN

As the protein sequence space grows, it becomes increasingly important to continuously improve and update natural classification of protein families. Makarova et al provide a unified classification for CRISP-associated (Cas) proteins and a scenario for the origins of CRISP-Cas. This work was carried out by employing a classical computational genomics approach based on analysis of protein sequences and structures. The subject of this paper is outside my immediate expertise and therefore I will not comment on its potential significance and impact for this field of CRISP-Cas systems and antiviral immunity in prokaryotes. Authors should rely on other reviewers in this regard. In my opinion, authors have provided essential background information about the systems and it was easy to follow the logic of their story. From the technical point of view, this is a well-executed study, which is not surprising considering authors' expertise and their standing in the field. However, the overall presentation is not as CRISP as the title might suggest. Papers presenting original research must provide enough detail, so results can be independently verified and reproduced, which is not the case here. This is primarily due to two issues that I have outlined below as major concerns.

Response: *We are perplexed by the blanket criticism of our work and strongly disagree. Some additional details are provided in response to the specific comments below, and a few unfortunate mistakes in the references to Additional Files are corrected. Overall, however, we maintain that the manuscript as submitted conformed to the presentation standards in the field of computational molecular biology. Furthermore, we see an internal contradiction in the reviewer's comment: it is either "a well-executed study, which is not surprising considering authors' expertise and their standing in the field" or "Papers presenting original research must provide enough detail, so results can be independently verified and reproduced, which is not the case here". It cannot be both ways*.

#### MAJOR CONCERNS

1. Data and procedures are not described adequately. I will illustrate this point by a few examples taken from the very first section of Results & Discussion (pages 7 and 8): a. Authors state (lines 127-128): "... we performed exhaustive sequence database searches [IZ: which database? See also comment 2b] for all Cas protein families using the HHpred profile against profile search method". Does it mean "using sequences of representative members of these families?" As far as I understood, HHpred input requires a sequence, not a protein family, or a multiple sequence alignment. Therefore, authors should provide a list of all sequences that were used as queries in these HHpred searches, explaining why they were chosen (e.g. experimentally confirmed function, structure available, was previously predicted to be a Cas protein, etc) and providing corresponding references. They also should present the search result for each sequence, including the E-value and reported probability to be a false positive, similarly to the example shown on lines 130-134. A simple table in Excel would be a great choice to show these input data and search results. As it stands, I can only see input data and results for two sequences: ST0029 (line 130) and its reciprocal hit (line 133).

Response: *The list of databases we used to search against using HHpred is provided in the Methods section. For "exhaustive" sequence database searches we usually run PSI-BLAST (and HHpred) with several diverged representatives within the family to ensure maximum coverage. It does not make any sense to report all these results because we present an alignment and use the results of multiple searches to manually correct it. This is not a fully automated process, and it will not be reproducible as soon as the databases change, and they change continuously*.

*For alignments we select representatives manually, again trying to cover the maximum diversity within the family both in terms sequence diversity and taxonomy, but do not include fragments or sequences which are disrupted in some way and/or contain long insertions or deletions because our aim here is to showcase the typical representatives of a family. We believe that all the alignments we provide give an adequate family representation to back all the claims made in the text*.

*We find the demands for greater level of detail (e.g., "*They also should present the search result for each sequence") *unreasonable and out of line with the de facto standards*.

b. Authors state (lines 134-137): "We used the alignments obtained during these and other searches started from other query sequences along with secondary structure prediction to construct multiple alignments for Cas7 and a number of most closely related RAMP subfamilies (Figure 1)". What other searches? What other query sequences? Neither the follow-up text nor the figure legend for Figure 1 provides answers to these questions. Why these and not other sequences are shown on Figure 1? For example, Figure 4A shows at least two Cas7 genes in *Staphylococcus epidermidis*, but no sequences from this organism can be found on Figure 1. So, my guess is that Figure 1 shows representative sequences, but again this is just my guess. If it is so, why these sequences were selected as representative (e.g. to illustrate diversity or only highly scoring sequences are shown)? I cannot find any explanation anywhere in this paper. I was able to find one sequence from Staphylococcus epidermidis in Additional File 1 (which presumably contains the most comprehensive data). In accordance with Figure 4A, this sequence is shown under Cas7 group. I have figured out this is the protein encoded by the second Cas7 gene from the cluster shown in Figure 4A (labeled as Csm5); however, there is no sequence for the protein encoded by the first Cas7 gene (Csm3) in the Additional File 1. Is this an error on Figure 4A or incomplete data representation in Additional File 1?

Response: *See the response to the previous comment. We have added the Csm3 protein from Staphylococcus epidermidis in the *Additional File 1 *but not to *Figure 1 *because Csm3 from Staphylococcus epidermidis is quite similar to the ortholog from Mycobacterium tuberculosis (Expect = 1e-37 Identities = 93/226 (41%), Positives = 132/226 (58%)) which is present in the *Figure 1 and we *can show only a limited number of sequences in the figure*.

c. Authors state (lines 149-150): "Examination of this structure unequivocally demonstrates the presence of a single RAMP domain...[16]". Even if we ignore the wording (although I maintain a view that examination cannot demonstrate anything), it is unclear who came up with this conclusion: authors of the current paper or those who published the structure, without reading the structure paper. The continuation of this description (lines 150-152) is also confusing: "...a single RAMP domain that contains four inserts within the RRM core and a C-terminal extension. None of these additional domains of Cas7 show sequence or structural similarity to any known domains [16]". Is this a direct quote from reference 16? What is meant by "these additional domains"? Are the four inserts within a domain core and a C-terminal extension referred to as domains? Why, if they show no sequence or structural similarity to any known domains???

Response: *for the sake of style '*unequivocally' *has been replaced with *'clearly'. *That apart, however, we find the text in question to be unequivocal. The 'examination' that, in our firm opinion, is quite capable of demonstrating things comes from Ref. 16 but we concur with their conclusions. This is perfectly clear from the text. The 'additional domains' are inserts, and this is clear as well*.

d. Authors state (lines 156-158): "We performed... HHpred searches using representatives of 19 RAMP families... (Additional File 1). What are these 19 families? I couldn't find any reference to them in the Background or in the Additional File 1, which contains over 30 individual MSAs and some of them are labeled as "family" (e.g. Cas6 family, the very last one), some are labeled as "subfamily" (e.g. Cas5 BF2549 subfamily), some are labeled as "group" (e.g. MTH323/Csm4 (Cas5 group)), and majority do not have labels that would identify them as a family (they have names, but are those family names?). Let us treat the first three lines of the Additional File 1 as its title (Please see TECHNICAL COMMENTS below regarding this file). It reads: "Cas7 group (COG1857 and other Cas7, COG1337/Csm3; COG1604/cmr6; COG1336/Cmr4; Csc2; Csf2; Csy3; Csm5 (COG1332), Cmr1 (COG1367, double Cas7). Let us assume that families are separated by comas and semicolons. Then there are 9 families in this group. Following the same assumption, there are 4 families in each of the Cas5 and Cas6 groups. I still cannot figure out what are the 19 families then. Perhaps, the first three lines of this file are not the title after all. This suspicion is confirmed by the main text (line 163), which lists 5 RAMPs in the Cas5 group (the 5th is Csy2, which is missing from the Cas5 description on the top of Additional File 1). The bottom line is that there is no place in the paper, where 19 families are clearly specified.

Response: *Unfortunately, in lines 156-158 of the original manuscript, we have mistakenly referred to a wrong Additional File. The correct reference is to *Additional File 3 *(all the 19 families in question are listed in *Additional File 3 *and their names correspond to the families with the same names in *Additional File 1*)*. Additional File 1 *also contains some other alignments most of which are relevant to the discussion of different RAMP families and CRISPR-Cas systems throughout the text. We believe that these alignments will be useful to researchers studying individual CRISPR-Cas systems because they cover all the RAMP diversity we aware of. The family names are given according to Makarova et al. *[20] *alternative family identifiers are given in parentheses. We modified the description of the families for the sake of greater consistency. Some explanations were added to the *Additional File 1 *header. The GI IDs of sequences that were used for the corresponding HHpred searches are now available in *Additional File 3.

e. Returning to the main text (line 158): "... and collected similarity scores between corresponding profiles (Additional File 1)". I cannot see any similarity scores or any profiles in this file or anywhere else. I have stopped evaluating the adequacy of presentation after page 8, because it will require too much time, but I hope that authors will continue the trend I have initiated here and critically reevaluate their data presentation.

Response: *Unfortunately, it was a wrong reference to Additional File. Corrected*.

2. Methods are described at the very minimum. This section (half a page) looks like a methods summary, not methods description.

Response: *Given that the methods used in this study are all published, the Methods section is generally adequate. A few details were added in response to the comments below. No one would gain anything from lengthy descriptions*.

a. For PSI-BLAST, two search parameters were specified (page 34, line738), whereas no other details provided for any other type of analysis. If all parameters were default, it should be specifically stated.

Response: *It is now stated in the Methods section*.

b. There is a lack of consistency (and detail) in describing database search tools and databases. In one case, a search tool is specified (PSI-BLAST), but the database(s) is not (page 34, line737); whereas in the other case a database is specified (CDD), but the search tool (RPS-BLAST) and its parameters are not (page 34, line 740). No database is specified for HHpred searches (page 34, line 741).

Response: *It is now stated in the Methods section*.

c. Authors state (lanes 741-743): "Following the recommendations of the HHpred authors, we used the reported probability of a true positive match rather than the e-value to assess the statistical significance of a hit [27]". After reading the reference 27, I didn't get a sense that probability of a true positive match should be used instead of the E-value. Soding indeed writes that "E-values reported by most tools, including ours, can be very unreliable", but my take is that he suggests using the probability of a true positive match in addition to and not instead of the E-value.

Response: *We read this statement of Söding as an indication of the superiority of the true positive match probabilities over the e-values because, unlike the theoretically computed e-values, these probabilities come from the analysis of a benchmarked set*.

In any case, at least one of these parameters must be provided for all search results (see comment 1a).

Response: *On this occasion, the purpose of the analysis is not to reinforce the relatedness of the RAMPs (which has been demonstrated previously with a variety of methods) but rather to provide a metric for comparison of different RAMP families. The only part of the HHpred output that is suitable for this purpose is the actual score, and accordingly*, Additional File 3 *includes the score values. Probability values are quoted in the text where appropriate*.

#### TECHNICAL COMMENTS

3. Additional File 1

a. What is shown in the Additional File 1? There is no title (I understand that the first 3 lines sort of serve the purpose of the title, but it is certainly not the title and they contain errors - see comment 1d) and no footnote explaining it.

Response: *The title of the file was provided in the list of all additional files included to the paper. The header for *Additional File 1 *is included in the revision. Explanations for the content of the file are also included*.

b. Were these alignments automatically generated? It looks like a raw output file from some program or server. Reference to this file on page 8 (lines 156-159) is confusing: authors state that this is as a result of both DALI and HHpred searches. How so? Were DALI outputs used as inputs for HHPred? Full explanation of what is shown in this file is needed.

Response: *The alignments were constructed as described in the Methods section. HHpred alignments are not included. HHpred probability values are reported in *Additional file 3. *Other explanations relevant to this concern are provided above*.

c. For some alignments, both 2D prediction and consensus are shown (e.g. Csy3), whereas for others only 2D prediction (e.g. Csb1, Cmr6) is shown. Is there any reason for that? No explanation is given to the fact that for some groups both 90% and 100% consensus are shown (e.g. Cmr4), whereas for others - only 85% consensus (e.g. Cas7 ygcJ). In some cases, results of 2D prediction and consensus are shown underneath the alignment (e.g. Csy3), whereas in other cases consensus is shown above and 2D underneath (e.g Cas7 (ygcJ). Amazingly, in some cases, 2D prediction is shown in the middle of the family alignment (e.g. Csb1). Is this all due to scripting errors? If so, how do we know whether in this case the upper part of the alignment is not in fact the bottom part of the alignment of another family shown above? This could have been a scripting error resulting in inserting a family id and some spacing in the middle of a family alignment... This inattention to the detail increases the probability of accidental errors and raises concerns.

Response: *In the revision, we show the 2D structure consistently underneath each alignment. The information on the program used to generate the consensus is provided in the header of *Additional File 1 *(the % of consensus was chosen ad hoc depending on the conservation with a family). Several additional consensus lines (for the families with a potential catalytic residues conserved) were included*.

## Reviewer's response

I regret that authors have interpreted my comments as "the blanket criticism of their work". Nowhere in my review have I questioned analyses, conclusions or the validity of their work.

My frustration with the lack of some key results (that apparently were present, but not in the file, which was referenced in the text) on the background of haziness in describing data collection and incompleteness in describing key methodologies, led me to strong wording regarding the reproducibility of results. In light of corrections and revisions made, I no longer maintain this view.

Although authors insisted that "the manuscript as submitted conformed to the presentation standards in the field of computational molecular biology", I am pleased to see that most of my requests for more detail and more attention to detail were accommodated in revision.

I believe that standards in such a new and rapidly evolving discipline as computational molecular biology should also evolve to reflect the unprecedented data growth. In reply to my requests for more transparency in describing results of database searches, authors wrote that "results will not be reproducible as soon as the database change, and they change continuously". I argue that exactly because the database content changes continuously, we must ensure results are reproducible. I understand that demanding each and every search detail will make research inefficient; however, there are relatively simple steps to ensure reproducibility of searches: working with (and specifying) a fixed dataset/database of known date/version, providing thresholds for searches and other settings for programs, and showing as many results of these searches as possible.

## Supplementary Material

Additional file 1 **Alignments of RAMP families**. Multiple sequence alignments supporting the classification of RAMPs into three groups. Click here for file

Additional file 2 **Topology diagrams of RAMPs with solved structure**. Topologies of the RRM domains in the RAMPs of Cas6 and Cas5 groups. Click here for file

Additional file 3 **Matrix of HHpred scores for RAMP families**. The Table shows pairwise HHpred scores for all subfamilies of RAMPs discussed in the text. Click here for file

Additional file 4 **Pattern of RAMPs of Cas7, Cas5 and Cas6 group in operons of different Type I and Type III systems**. Pattern of RAMPs in the CRISPR/Cas operons. Click here for file

Additional file 5 **Alignment of Cas10 and large subunits of Type I CRISPR-Cas systems**. The multiple alignments are presented as evidence of potential homology between large subunits of Type I and Type III systems and alpha-helical extensions present in several Cas8 families. Click here for file

Additional file 6 **Alignment of Cas9 and homologs**. This file contains multiple alignments of Cas9-related families and the homologs that share several domains with Cas9 but are not associated with CRISPR-Cas systems. The list of these Cas9 homologs in also provided. Click here for file

Additional file 7 **Proposed new names for *cas *gene families that currently have legacy names**. This file contains a table with new proposed "numbered" assignments for *cas *genes. Click here for file
