# Supplementary figures and images for: Unification of Cas protein families and a simple scenario for the origin and evolution of CRISPR-Cas systems
Source: Biol Direct. 2011 Jul 14;6:38. doi: 10.1186/1745-6150-6-38 (PMC3150331; doi:10.1186/1745-6150-6-38)

**1WJ9** Cas6e

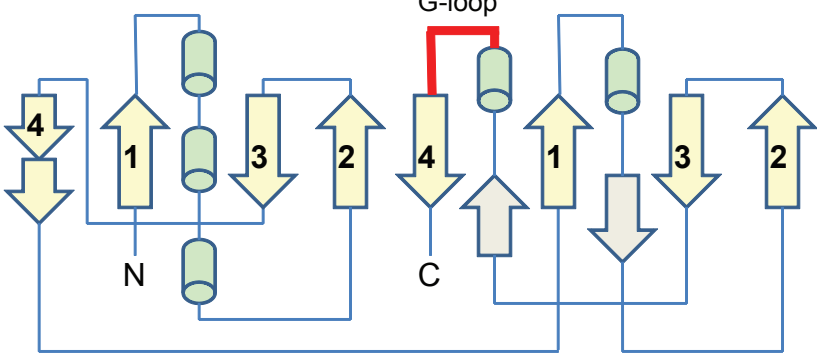

**1XLJ** Cas6f

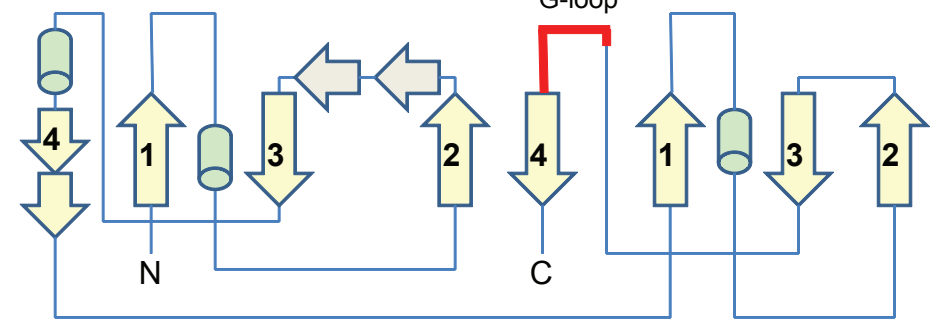

**3KG4** Cas5

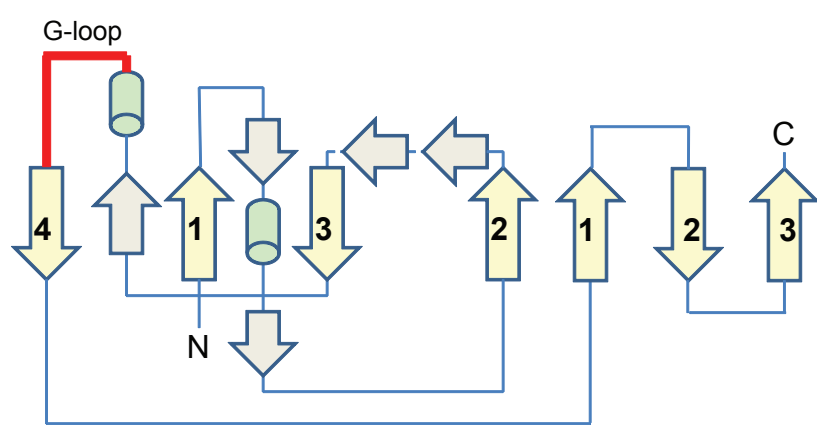

Supplement: Additional file 2 — Topology diagrams of RAMPs with solved structure. Topologies of the RRM domains in the RAMPs of Cas6 and Cas5 groups. [file 1745-6150-6-38-S2.PDF]
