# Supplementary material for: Unification of Cas protein families and a simple scenario for the origin and evolution of CRISPR-Cas systems
Source: Biol Direct. 2011 Jul 14;6:38. doi: 10.1186/1745-6150-6-38 (PMC3150331; doi:10.1186/1745-6150-6-38)
Supplement: Additional file 4 — Pattern of RAMPs of Cas7, Cas5 and Cas6 group in operons of different Type I and Type III systems. Pattern of RAMPs in the CRISPR/Cas operons. [file 1745-6150-6-38-S4.PDF]

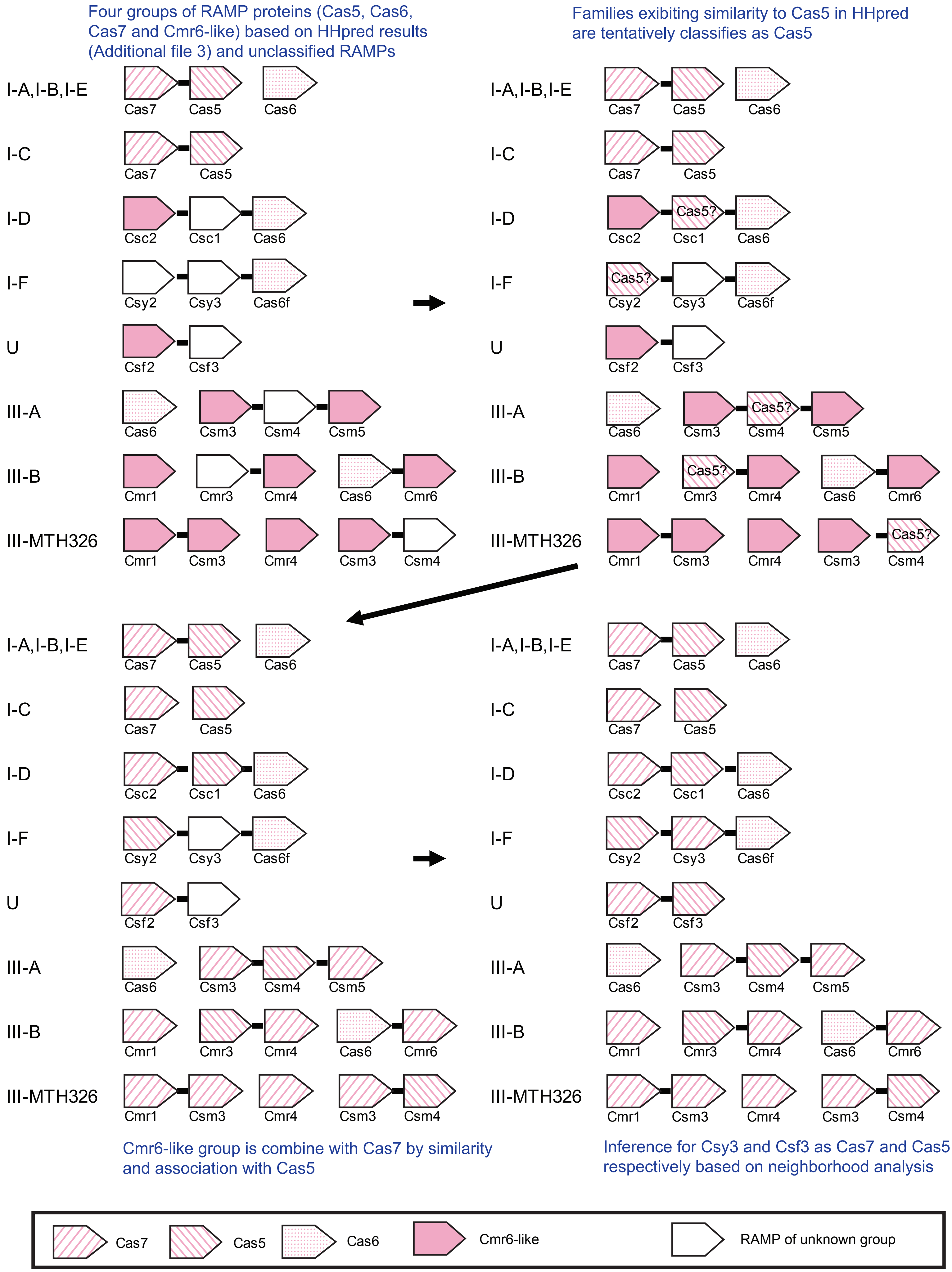
Four groups of RAMP proteins (Cas5, Cas6, Cas7 and Cmr6-like) based on HHpred results (Additional file 3) and unclassified RAMPs
Families exibiting similarity to Cas5 in HHpred are tentatively classifies as Cas5
I-A,I-B,I-E
Cas7 Cas5 Cas6
I-C
Cas7 Cas5
I-D
Csc2 Csc1 Cas6
I-F
Csy2 Csy3 Cas6f
U
Csf2 Csf3
III-A
Cas6 Csm3 Csm4 Csm5
III-B
Cmr1 Cmr3 Cmr4 Cas6 Cmr6
III-MTH326
Cmr1 Csm3 Cmr4 Csm3 Csm4
Cas5?
Cmr6-like group is combine with Cas7 by similarity and association with Cas5
Inference for Csy3 and Csf3 as Cas7 and Cas5 respectively based on neighborhood analysis
Cas7
Cas5
Cas6
Cmr6-like
RAMP of unknown group
